# Mice deficient in the lysosomal enzyme palmitoyl-protein thioesterase 1 (PPT1) display a complex retinal phenotype

**DOI:** 10.1038/s41598-019-50726-8

**Published:** 2019-10-02

**Authors:** Yevgeniya Atiskova, Susanne Bartsch, Tatyana Danyukova, Elke Becker, Christian Hagel, Stephan Storch, Udo Bartsch

**Affiliations:** 1 Department of Ophthalmology, Experimental Ophthalmology, University Medical Center Hamburg-Eppendorf, Hamburg, Germany; 2 Children´s Hospital, Department of Biochemistry, University Medical Center Hamburg-Eppendorf, Hamburg, Germany; 3 Institute of Neuropathology, University Medical Center Hamburg-Eppendorf, Hamburg, Germany; 4 Present Address: Department of Osteology and Biomechanics, University Medical Center Hamburg-Eppendorf, Hamburg, Germany

**Keywords:** Experimental models of disease, Experimental models of disease, Neurodegeneration, Neurodegeneration

## Abstract

Neuronal ceroid lipofuscinosis (NCL) type 1 (CLN1) is a neurodegenerative storage disorder caused by mutations in the gene encoding the lysosomal enzyme palmitoyl-protein thioesterase 1 (PPT1). CLN1 patients suffer from brain atrophy, mental and motor retardation, seizures, and retinal degeneration ultimately resulting in blindness. Here, we performed an in-depth analysis of the retinal phenotype of a PPT1-deficient mouse, an animal model of this condition. Reactive astrogliosis and microgliosis were evident in mutant retinas prior to the onset of retinal cell loss. Progressive accumulation of storage material, a pronounced dysregulation of various lysosomal proteins, and accumulation of sequestosome/p62-positive aggregates in the inner nuclear layer also preceded retinal degeneration. At advanced stages of the disease, the mutant retina was characterized by a significant loss of ganglion cells, rod and cone photoreceptor cells, and rod and cone bipolar cells. Results demonstrate that PPT1 dysfunction results in early-onset pathological alterations in the mutant retina, followed by a progressive degeneration of various retinal cell types at relatively late stages of the disease. Data will serve as a reference for future work aimed at developing therapeutic strategies for the treatment of retinal degeneration in CLN1 disease.

## Introduction

Neuronal ceroid lipofuscinosis (NCL) comprise a genetically and clinically heterogeneous group of lysosomal storage disorders that is characterized by an intracellular accumulation of autofluorescent storage material, progressive neurodegeneration and premature death. Although it is a rare disease with an estimated incidence of 1:14,000 to 1:67,000 depending on the ethnic group and founder group effects, NCLs constitute the major group of neurodegenerative diseases in childhood^1–3^. Originally classified mainly according to the age at disease onset, the different NCLs are now grouped according to the affected gene (CLN1-CLN8 and CLN10-CLN14)^4–7^. Patients present with brain atrophy, mental and motor retardation, and epileptic seizures, and die prematurely. Retinal degeneration resulting in visual impairment and ultimately blindness is another characteristic clinical feature of most NCLs^6,8–12^.

NCL type 1 (CLN1) disease, also termed infantile neuronal ceroid lipofuscinosis (INCL) or infantile Batten disease, is caused by mutations in the *PPT1* gene on chromosome 1p34.2^13^. Until now, 71 mutations and 9 polymorphisms have been identified in the *PPT1* gene [https://www.ucl.ac.uk/ncl-disease/] which encodes the soluble lysosomal enzyme palmitoyl-protein thioesterase 1 (PPT1). The enzyme is involved in the lysosomal degradation of lipid-modified proteins by removing thioester-linked fatty acyl groups from cysteine residues, and has been implicated in synaptogenesis, regulation of synaptic vesicle endo- and exocytosis, endosomal trafficking and lipid metabolism^5,7,8^. While PPT1 is ubiquitously expressed, high PPT1 enzymatic activity has been shown in the brain and particularly in the retina^14,15^.

Clinically, CLN1 disease is characterized by an early-onset and rapidly progressing cerebral atrophy resulting in deterioration of cognitive and motor functions^11,16–19^. Mental retardation becomes obvious at about 9–19 months of age, and is accompanied by an early loss of motor functions resulting in hypotonia, ataxia and myoclonic jerks^11,19,20^. Epileptic seizures usually occur at advanced stages of the disease, and patients die at about 10 years of age^20,21^. In addition to this most prevalent CLN1 disease ‘classic infantile NCL’ (INCL) variant, patients harboring mutations in the *PPT1* gene might develop first clinical symptoms later in life, and present with a more slowly progressing late-infantile, juvenile or adult onset NCL^22–28^.

Retinal degeneration resulting in vision loss is another hallmark of CLN1 disease, and visual impairment is usually diagnosed at about 12–22 months of age^19^. Ophthalmic examinations demonstrated maculopathy with pigmentary alterations, atrophy of the optic nerve and retinal vessel attenuation^11,16,17^. Furthermore, electroretinogram (ERG) recordings revealed an early-onset and rapidly progressing deterioration of visual function^16,17,29^. Histological analyses of the retina of a CLN1 patient at a late stage of the disease revealed autofluorescent storage material throughout the retina, and significant loss of photoreceptor cells, neurons of the inner nuclear layer, and ganglion cells^17^. In comparison, CLN1 patients with a later onset of the disease presented with night blindness and moderate visual impairment as juveniles, resulting in progressive vision loss and extinguished ERGs at later ages^23,27,28^. A slowly progressing visual impairment was also observed in CLN1 patients with an adult onset of the disease. Deterioration of visual function was accompanied by no or only mild funduscopic abnormalities, such as pallor of the optic nerve and pigmentary alterations of the macula^25,26^.

Several transgenic mouse models of CLN1 disease have been generated, and all have been shown to faithfully recapitulate many of the pathological features observed in CLN1 patients, such as accumulation of autofluorescent granular osmiophilic deposits in neural and visceral tissues, rapidly progressing neurodegeneration in the brain, motor abnormalities, seizures and premature death^30–33^. Analyses of the retina of these mutants demonstrated accumulation of storage material, thinning of different retinal layers, and loss of photoreceptor cells and retinal ganglion cells. In addition, behavioral tests and ERG recordings have demonstrated progressive deterioration of retinal function^31,32,34–36^. However, detailed information about the progression of retinal degeneration, the molecular changes associated with the retinal pathology, and the impact of PPT1 deficiency on specific retinal neurons, such as rod and cone photoreceptor cells or specific retinal interneurons, is limited. In the present study, we therefore performed an in-depth analysis of the retinal phenotype of a *Ppt1* knock-out (ko) mouse. Specifically, we performed biochemical and ultrastructural analyses of the storage material, analyzed the expression of various lysosomal proteins, and quantified retina thinning and the loss of different retinal cell types during the course of the disease. Results of the present study will serve as a reference for future work aimed at establishing therapeutic strategies for the treatment of retinal degeneration in CLN1 disease.

## Material and Methods

### Animals

*Ppt1* ko mice^30^ and wild-type mice were maintained on a C57BL/6J genetic background and housed under standard conditions in the specific pathogen-free animal facility of the University Medical Center Hamburg-Eppendorf (Hamburg, Germany). For both genotypes male and female mice were included in the analyses, with no significant differences of the obtained results between sexes. All animal experiments were approved by the ‘Freie und Hansestadt Hamburg, Behörde für Gesundheit und Verbraucherschutz’ (reference number: ORG842) and were in accordance with the EU Directive for animal experiments.

### Immunohistochemistry and electron microscopy

Immunohistochemical analyses of mutant retinas were performed as described^37–39^. In brief, *Ppt1* ko and wild-type mice were sacrificed at the age of 45, 112 or 240 days, and eyes were fixed in 4% paraformaldehyde in phosphate buffered saline (PBS), cryoprotected and frozen. Cryostat sections, 25 µm in thickness, were blocked in PBS containing 0.1% bovine serum albumin (BSA) and 0.3% Triton X-100 (TX-100; both from Sigma-Aldrich, Deisenhofen, Germany), incubated with primary antibodies (Table 1), washed in PBS, and incubated with Cy2- or Cy3-conjugated secondary antibodies (Jackson Immunoresearch Laboratories, West Grove, PA, USA). Cone photoreceptor cells were labelled with biotinylated peanut agglutinin (PNA; 1:5,000; Vector Laboratories, Burlingame, CA, USA) followed by Cy3-conjugated streptavidin (1:500; Jackson Immunoresearch Laboratories). Sections were stained with 4′,6-diamidino-2-phenylindole (DAPI; Sigma-Aldrich), washed, and mounted onto slides. Retinal ganglion cells (RGCs) were additionally visualized in retina flatmounts using antibodies to brain-specific homeobox/POU domain protein-3A (BRN-3A) as described^40^. Retinal sections and flatmounts from *Ppt1* ko and age-matched wild-type mice were processed in parallel for each antigen to allow for direct comparison of staining intensities. At least six animals were analyzed for each antigen, genotype and age. For qualitative documentation, z-stacks were taken from central retinal regions close to the optic disc with an AxioObserverZ.1 microscope equipped with an ApoTome.2 (Zeiss, Oberkochen, Germany) using identical microscope settings for each antigen. Ultrastructural analyses of retinas from 240-day-old *Ppt1* ko and wild-type mice (n = 3 for each genotype) were performed as described^37,38^.

**Table 1:** Primary antibodies.

Antigen	Dilution	Company/Reference	Catalog Number
brain-specific homeobox/POU domain protein 3A (BRN-3A); (IHC)	1:200	Santa Cruz Biotechnology Inc., Santa Cruz, CA, USA	Sc-31984
cathepsin D (CTSD); (IHC, WB)	1:100 (IHC) 1:1000 (WB)	R&D Systems GmbH, Wiesbaden, Germany	AF1029
cathepsin X/Z/P (CTSZ); (IHC, WB)	1:100 (IHC) 1:1000 (WB)	R&D Systems GmbH	AF1033
cluster of differentiation 68 (CD68); (IHC)	1:1000	Bio-Rad-Laboratories, Kidlington, UK	MCA1957
glycerinaldehyde 3-phosphate dehydrogenase (GAPDH); (WB)	1:1000	Santa Cruz Biotechnology Inc.	Sc-25778
glial fibrillary acidic protein (GFAP); (IHC)	1:500	Dako Cytomation GmbH, Hamburg, Germany	Z0334
ionized calcium-binding adapter molecule 1 (IBA1); (IHC)	1:200	Wako Chemicals GmbH, Neuss, Germany	019–19741
lysosomal-associated membrane protein 1 (LAMP1); (IHC, WB)	1:2000 (IHC) 1:250 (WB)	Developmental Studies Hybridoma Bank, Iowa City, IA, USA	Clone 1D4B
lysosomal-associated membrane protein 2 (LAMP2); (IHC, WB)	1:200 (IHC) 1:250 (WB)	Developmental Studies Hybridoma Bank	Clone ABL-93
protein kinase C alpha (PKCα); (IHC)	1:500	Santa Cruz Biotechnology Inc.	Sc-208
saposin D; (IHC, WB)	1:2000	Konrad Sandhoff, Bonn, Germany^94^	
secretagogin (SCGN); (IHC)	1:2000	BioVendor Research and Diagnostic Products, Brno, Czech Republic	RD184120100
sequestosome 1/p62 (SQSTM1/p62); (IHC)	1:1000	Enzo Life Sciences, Lausen, Switzerland	BML-PW9860
SQSTM1/p62; (WB)	1:1000	Cell Signaling, Danvers, MA, USA	5114
subunit c of mitochondrial ATP synthase; (IHC)	1:1000	Abcam, Cambridge, UK	ab181243
anti-α-tubulin (WB)	1:5000	Sigma-Aldrich, Deisenhofen, Germany	T9026

IHC: immunohistochemistry, WB: Western blot.

### Determination of retina thickness and retinal nerve cell numbers

Optical sections with a thickness of 0.25 µm were taken from the nasal to the temporal periphery of central (i.e. in the plane of the optic disc) retinal sections using an AxioObserverZ.1 microscope equipped with an ApoTome.2. The thickness of the entire retina (i.e. from the retinal pigment epithelium to the vitreal margin of the retina), the inner retina (i.e. from the outer plexiform layer to the vitreal margin of the retina) and the inner nuclear layer was measured at nine equidistant positions of both the nasal and temporal retina. Rows of photoreceptor cell nuclei were counted at the same positions. PNA-labeled cone photoreceptor cells and BRN-3A-positive RGCs were counted over the entire length of central retinal sections. Cones were only counted when their inner segments were in direct contact with the outer nuclear layer. RGCs were counted provided they had a clearly visible DAPI-stained nucleus. Numbers of BRN-3A-positive RGCs were additionally determined in retinal flatmounts from 240-day-old animals as described^40^. The density of protein kinase C alpha (PKCα)-positive rod bipolar cells and secretagogin (SCGN)-positive cone bipolar cells with clearly visible DAPI-labeled nuclei was determined in three equidistant areas each with a length of 250 µm in both the nasal and temporal retinal half. GFAP-positive Müller cell processes that crossed a line at half height of the inner plexiform layer were counted in nasal and temporal retinal halfs in areas (500 µm in width) located midway between the optic disc and retinal periphery. The mean gray value and the integrated density (i.e. mean gray value x immunoreactive area) of the GFAP immunostaining signal in retinal astrocytes was determined in the same retinal areas using ImageJ software (Rasband, W.S., ImageJ, U. S. National Institutes of Health, Bethesda, Maryland, USA). Mean gray values and integrated density values of the GFAP fluorescence were compared between mutant and age-matched wild-type retinas that were processed in the same immunostainings and photographed using the same microscope settings. Six *Ppt1* ko mice and six wild-type mice were analyzed for each antigen and age. Finally, we determined the percentage of CD68-positive cells showing strong immunoreactivity for CTSD or CTSZ and of IBA1-positive cells showing strong immunoreactivity for CTSD, CTSZ, LAMP1 or LAMP2 in z-stacks through the entire thickness of retina sections (25 µm) from P240 *Ppt1* ko retinas (n = 6). Countings were done in the nasal and temporal retinal half in areas (500 µm width) located midway between optic disc and retinal periphery. All quantitative analyses were performed in a blinded fashion on number-coded retinal sections and flatmounts. Statistical analyses of cell counting and thickness measurements were performed using the two-way ANOVA and three-way ANOVA, respectively, followed by a Bonferroni post-hoc test. Statistical analyses of ganglion cell densities in retinal flatmounts were done with the Mann-Whitney U test. Mean intensity and integrated density values of the GFAP immunostaining signal in retinal astrocytes were analyzed using the paired Student’s t-test.

### Retinal protein extraction and western blotting

Total protein homogenates were prepared from 240-day-old *Ppt1* ko and age-matched control retinas by suspension in 200 µl ice-cold lysis buffer (50 mM Tris-HCl, pH 7.4, 0.15 M NaCl, 1% TX-100 supplemented with protease inhibitors) followed by incubation of protein extracts for 30 min on ice. After centrifugation at 16,000xg for 10 min, protein concentrations of supernatants were determined using Bradford protein assay dye reagent (Bio-Rad, Munich, Germany) and BSA (Thermo Fisher Scientific, Schwerte, Germany) as a standard.

Extraction of proteins from retinas for the detection of prosaposin and saposin D by immunoblotting was performed as described^41^. For the detection of sequestosome 1/p62 (SQSTM1/p62), retinas were homogenized in 200 µl 50 mM Tris-HCl (pH 7.4) containing protease inhibitors. After addition of 50 µl 10% SDS (final concentration: 2%), homogenates were sonicated for three times using a microtip sonifier and cleared by centrifugation for 10 min at 16.000xg. Protein concentrations of supernatants were determined using the bicinchoninic acid assay (Roth, Karlsruhe, Germany). Aliquots of total protein extracts (30 µg) were separated by SDS-PAGE, blotted onto nitrocellulose membranes and processed for immunoblotting. Immunoreactive bands were visualized using enhanced chemiluminescence reagent (Thermo Fisher Scientific) and quantified using the ChemiDoc XRS system and Quantity One software (Bio-Rad). Molecular masses of immunoreactive proteins were determined by comparison with the electrophoretic mobility of prestained standard proteins (Page Ruler™, Thermo Fisher Scientific). Each experiment was performed in triplicate on retinal extracts from different *Ppt1* ko and age-matched control mice. Statistical analyses of data were performed using the Student’s *t*-test.

## Results

In the present study, we performed an in-depth analysis of the retinal phenotype of a PPT1-deficient mouse, an animal model of CLN1 disease. Using immunohistochemistry, morphometry, electron microscopy and immunoblot analyses we provide data about the onset and the progression of the retinal dystrophy, and the molecular and cellular changes associated with the retinal pathology.

### Early-onset retinal pathology in the *Ppt1* ko mouse

**Figure 1:**
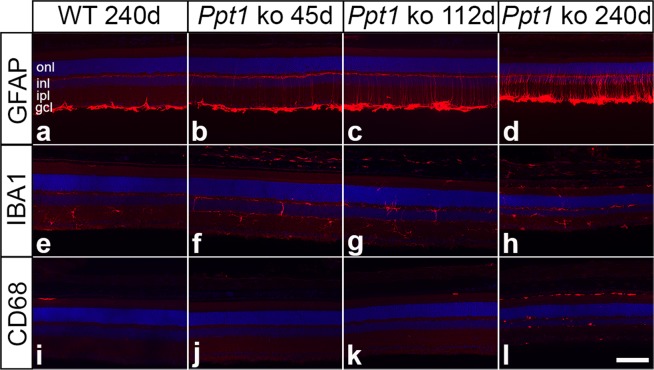
Reactive astrogliosis and microgliosis in PPT1-deficient retinas. Expression of GFAP, IBA1 and CD68 in wild-type and *Ppt1* ko retinas. Note the presence of some faintly GFAP-positive Müller cells in mutant retinas at P45 **(b**). The number of positive Müller cells and expression levels of GFAP increased considerably with increasing age of the mutants (**c,d**). IBA-1-positive microglial cells were restricted to the inner retina of mutant mice at P45 (**f**), but became detectable in the outer retina and subretinal space at P112 (**g**) and P240 (**h**). A few CD68-positive cells were present in the outer retina of 112-day-old mutants (**k**), and their number was considerably increased in P240 *Ppt1* ko retina, particularly in the subretinal space (**l**). Immunostainings of 240-day-old wild-type retinas are shown for comparison (**a,e,i**). gcl: ganglion cell layer; inl: inner nuclear layer; ipl: inner plexiform layer; onl: outer nuclear layer. Scale bar in (**l**) (for **a–l**): 100 µm.

To determine the onset of the retinal pathology in the *Ppt1* ko mouse, we analyzed the expression of glial fibrillary acidic protein (GFAP), ionized calcium-binding adapter molecule 1 (IBA1) and cluster of differentiation 68 (CD68) in central retinal sections of mutant and wild-type mice at different ages. Expression of GFAP in wild-type mice was detectable in retinal astrocytes and a few Müller cells at all ages analyzed (for a 240-day-old wild-type retina, see Fig. 1a). In *Ppt1* ko retinas, in comparison, the number of GFAP-positive Müller cells was significantly increased at P45, P112 and P240 when compared with age-matched control retinas (Fig. 1b–d, Supplementary Fig. 1a; p < 0.001 for all comparisons; two-way ANOVA followed by a Bonferroni post-hoc test). Furthermore, the mean intensity of the GFAP immunostaining signal in retinal astrocytes of *Ppt1* ko mice was slightly but significantly elevated at P45 and P112 (p < 0.05 for both comparisons, paired Student’s t-test), and strongly increased at P240 (p < 0.001) when compared with age-matched wild-type mice (Fig. 1, Supplementary Fig. 1b). Integrated density values for the GFAP immunostaining signal in retinal astrocytes were similar between wild-type and *Ppt1* ko retinas at P45, but significantly increased in mutant retinas at P112 and P240 when compared to age-matched control retinas (p < 0.001 for both comparisons; Supplementary Fig. 1c). Together, data demonstrate an early-onset reactive astrogliosis in the retina of PPT1-deficient mice.

Cell bodies of IBA1-positive microglia cells were restricted to the inner retina (Fig. 1f), while CD68-positive cells were virtually absent from mutant retinas at P45 (Fig. 1j). At this age, some IBA1-positive cells in mutant retinas, but not in wild-type retinas, extended processes into the outer nuclear layer. In 112-day-old *Ppt1* ko retinas, a few IBA1- (Fig. 1g) and CD68-positive cells (Fig. 1k) became additionally detectable in the outer nuclear layer and in the subretinal space, and their number increased considerably until P240, particularly in the subretinal space (Fig. 1h,l). In wild-type mice, in comparison, subretinally located IBA1- or CD68-positive microglia/macrophages were not detected in young animals, and were only rarely observed in 240-day-old mice (Fig. 1e,i).

### Accumulation of storage material

**Figure 2:**
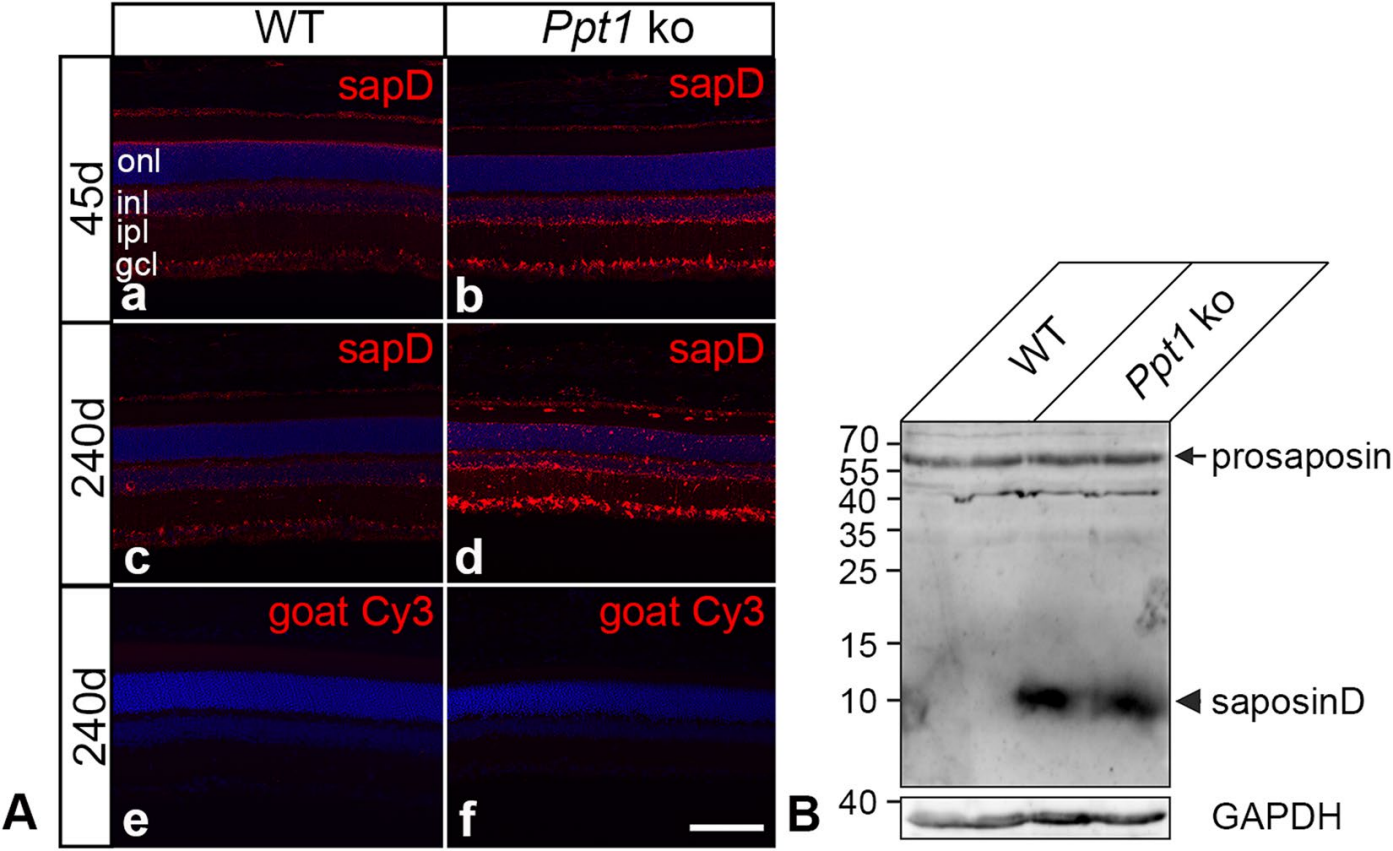
Accumulation of Saposin D in Ppt1 ko retinas. (**A**) Immunohistochemical analyses revealed moderately and markedly elevated levels of saposin D in 45- (b) and 240-day-old *Ppt1* ko retinas (d) respectively when compared to age-matched wild-type retinas (a and c, respectively). No fluorescence signal was detectable when wild-type (e) or mutant (f) retinal sections were incubated with secondary antibodies only. (**B**) Immunoblot analyses confirmed markedly increased levels of saposin D (arrowhead) in 240-day-old mutant retinas, and revealed similar levels of prosaposin (arrow) in both genotypes. GAPDH immunoblotting was performed as a loading control. Positions of molecular mass markers are indicated. Uncropped blots are shown in Supplementary Fig. 5. gcl: ganglion cell layer; inl: inner nuclear layer; ipl: inner plexiform layer; onl: outer nuclear layer. Scale bar in (f) (for a–f): 100 µm.

**Figure 3:**
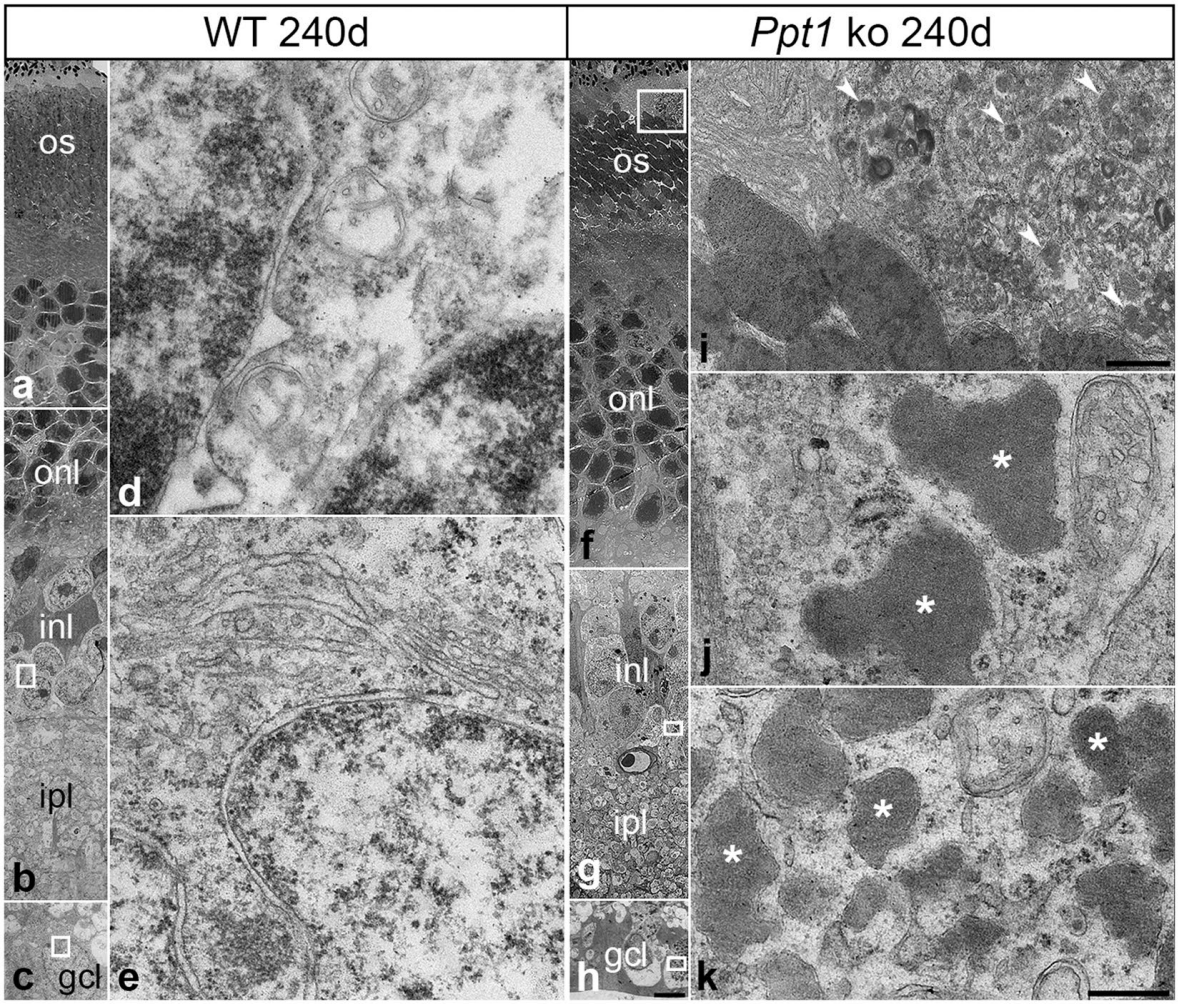
Ultrastructural analysis of storage material in the retina of Ppt1 ko mice. Low-power electron micrographs depict the different layers of a 240-day-old wild-type (**a–c**) and age-matched *Ppt1* ko retina (**f–h**). Analyses of the boxed areas in (**f–h**) at higher magnification revealed the presence of granular osmiophilic deposits in subretinally located macrophages (arrowheads in **i**), retinal interneurons (asterisks in **j**) and retinal ganglion cells (asterisks in **k**) respectively of *Ppt1* ko retinas. Similar cytoplasmic inclusions were not detectable in retinas of age-matched control mice (**d,e**). gcl: ganglion cell layer; inl: inner nuclear layer; ipl: inner plexiform layer; onl: outer nuclear layer; os: outer segments. Scale bar in (**h**) (for **a–c,f–h**): 5 μm; in (**i**): 1 μm; in (**k**) (for **d,e,j,k**): 0.2 μm.

A hallmark of NCL is the accumulation of autofluorescent storage material composed of low molecular weight hydrophobic proteins, mainly subunit c of mitochondrial ATP synthase and/or sphingolipid activator proteins (saposins) A and D^42,43^. Immunostainings revealed slightly elevated levels of saposin D in P45 mutant retinas (Fig. 2Ab) when compared to age-matched wild-type retinas (Fig. 2Aa). At this age, elevated levels of saposin D were mainly detected in the ganglion cell layer and inner nuclear layer of *Ppt1* ko retinas. At P240 saposin D-immunoreactivity was markedly increased throughout the mutant retina, and was particularly pronounced in the ganglion cell layer and in microglia/macrophages (compare Fig. 2Ad with Ac). Retinal sections incubated with the secondary antibody only were negative (Fig. 2Ae,Af). Immunoblot analyses confirmed the immunohistochemical observations, and revealed markedly increased levels of saposin D in 240-day-old *Ppt1* ko retinas when compared to age-matched control retinas (Fig. 2B). Levels of prosaposin, in contrast, were not significantly different between genotypes (Fig. 2B). At the ultrastructural level, we found electron-dense storage material in all cell types of the mutant retina, including photoreceptor cells, retinal interneurons and retinal ganglion cells (Fig. 3f–k). Similar electron-dense structures were not apparent in wild-type retinas (Fig. 3a–e). Storage material was particularly pronounced in subretinally located microglia/macrophages, and displayed the typical ultrastructure of granular osmiophilic deposits (Fig. 3f–k, Supplementary Fig. 2).

### Dysregulation of lysosomal proteins

**Figure 4:**
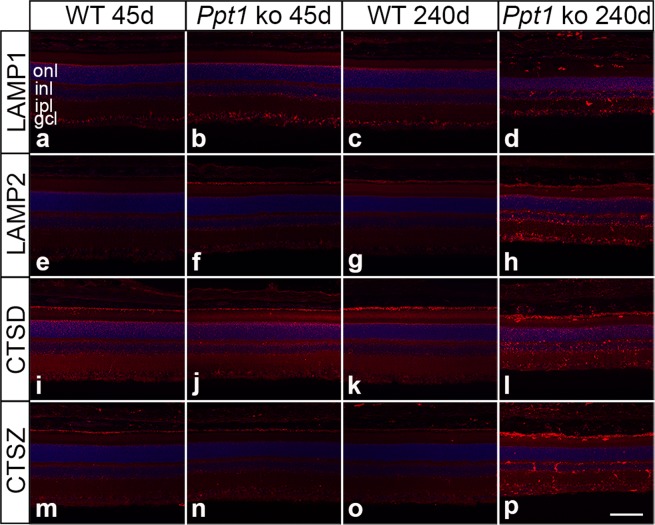
Dysregulation of lysosomal proteins in PPT1-deficient retinas. Expression levels of LAMP1, LAMP2, CTSD and CTSZ were slightly elevated in 45-day-old mutant animals (**b,f,j,n**, respectively) when compared to age-matched wild-type mice (**a,e,i,m**, respectively). Pronounced upregulation of LAMP1-, LAMP2-, CTSD- and CTSZ-immunoreactivity was observed in 240-day-old *Ppt1* ko retinas (**d,h,l,p**, respectively). The corresponding immunostainings of P240 control retinas are shown in (**c,g,k,o**), respectively. gcl: ganglion cell layer; inl: inner nuclear layer; ipl: inner plexiform layer; onl: outer nuclear layer. Scale bar in (**p**) for (**a–p**): 100 µm.

The impact of PPT1 deficiency on expression levels of various lysosomal proteins was studied by immunohistochemistry and immunoblot analyses. Immunostainings revealed slightly elevated levels of lysosomal-associated membrane protein 1 (LAMP1), lysosomal-associated membrane protein 2 (LAMP2) and the soluble lysosomal enzymes cathepsin D (CTSD) and cathepsin X/Z/P (CTSZ) in mutant retinas at P45 (Fig. 4b,f,j,n) when compared to age-matched wild-type retinas (Fig. 4a,e,i,m). Moderately and markedly elevated expression levels of these lysosomal proteins were detected in *Ppt1* ko mice at P112 (not shown) and P240 (Fig. 4d,h,l,p) respectively when compared to control mice (Fig. 4c,g,k,o). In 240-day-old mutant retinas, LAMP1-, LAMP2-, CTSD- and CTSZ-immunoreactivity was particularly pronounced in microglia/macrophages (Supplementary Fig. 3a–f″). Quantitative analyses revealed strong CTSD- and CTSZ-immunoreactivity in 97.9 ± 1.4% (mean ± SEM) and 96.7 ± 1.6% respectively of CD68-positive cells (Supplementary Fig. 3g). Furthermore, we found pronounced immunoreactivity for CTSD, CTSZ, LAMP1 and LAMP2 in 69.9 ± 1.7%, 82.4 ± 2.8%, 75.1 ± 2.3% and 67.9 ± 4.5% respectively of IBA1-positive cells (Supplementary Fig. 3g).

**Figure 5:**
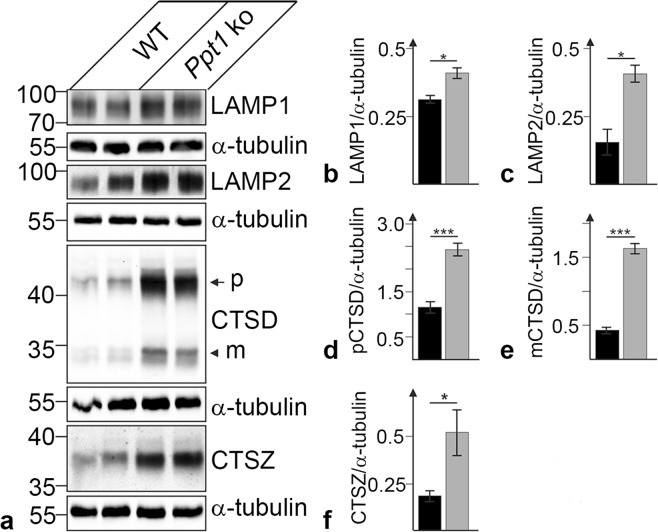
Dysregulation of lysosomal proteins in Ppt1 ko retinas. Immunoblot analyses revealed elevated levels of LAMP1, LAMP2, CTSD and CTSZ in 240-day-old *Ppt1* ko retinas when compared to age-matched wild-type retinas (**a**). α-tubulin immunoblotting was performed as a loading control. Positions of the molecular mass markers and the precursor (**p**) and mature (m) form of CTSD are indicated (**a**). Quantitative analyses of immunoblots demonstrated significantly increased levels of LAMP1 (**b**), LAMP2 (**c**), the precursor (**d**) and mature (**e**) form of CTSD, and CTSZ (**f**). Bars represent mean values (±SEM) of protein levels from three independent experiments normalized to the amounts of α-tubulin in 240-day-old wild-type (filled bars) and age-matched *Ppt1* ko retinas (grey bars). Uncropped blots are shown in Supplementary Fig. 5. *p < 0.05; ***p < 0.001 according to the Student’s t-test.

In agreement with the immunohistochemical data, immunoblot analyses revealed significantly elevated levels of LAMP1, LAMP2, CTSD and CTSZ in mutant retinas at P240 when compared to age-matched wild-type retinas (Fig. 5). Quantitative analyses of the immunoblots revealed that expression levels in *Ppt1* ko retinas were increased 1.3-fold for LAMP1 (p < 0.05; Student’s t-test), 2.6-fold for LAMP2 (p < 0.05), 2.1-fold for the CTSD precursor protein (p < 0.01), 3.9-fold for mature CTSD (p < 0.01), and 2.8-fold for CTSZ (p < 0.05) when compared to age-matched wild-type retinas (Fig. 5b–f).

### Regionally restricted expression of SQSTM1/p62 in *Ppt1* ko retinas

**Figure 6:**
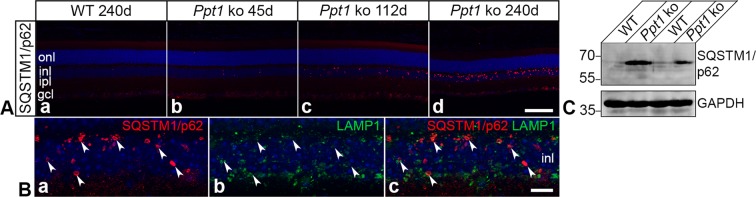
Expression of SQSTM1/p62 in the retina of Ppt1 ko mice. (**A**) SQSTM1/p62-positive aggregates were detectable in the retina of 45-day-old mutants (Ab) and increased in number and size with increasing age of the animals (Ac,Ad). Note that the immunoreactive punctae were restricted to the inner nuclear layer at all ages analyzed (Ab–Ad). Wild-type retinas were devoid of SQSTM1/p62-positive punctae (for a 240-day-old animal, see Aa). (**B**) There was either no or only partial co-localization of SQSTM1/p62-positive aggregates (Ba) with LAMP1 (Bb) in P240 *Ppt1* ko retinas (arrowheads in Ba–Bc). (**C**) Immunoblot analyses of retinas at P240 confirmed markedly elevated levels of SQSTM1/p62 in mutant retinas when compared to control retinas. GAPDH immunoblotting was performed as a loading control. Positions of the molecular mass markers are indicated. Uncropped blots are shown in Supplementary Fig. 5. gcl: ganglion cell layer; inl: inner nuclear layer; ipl: inner plexiform layer; onl: outer nuclear layer. Scale bar in (Ad) for (Aa–Ad): 100 µm; in (Bc) for (Ba–Bc): 10 µm.

Expression of the autophagy marker SQSTM1/p62 was not detectable in wild-type retinas at any age analyzed (for a P240 control retina, see Fig. 6Aa). In the mutant, in contrast, a few SQSTM1/p62-positive punctae were detectable at P45 (Fig. 6Ab), and their density increased considerably with increasing age of the mutants (Fig. 6Ac,Ad). Of note, SQSTM1/p62-positive aggregates were restricted to the inner nuclear layer of mutant retinas at all ages analyzed. Furthermore, double immunostainings revealed hardly any co-localization of SQSTM1/p62 with the lysosomal marker LAMP1 (Fig. 6Ba–Bc). Immunoblot analyses of retinas from P240 mutant and wild-type mice confirmed significantly elevated amounts of SQSTM1/p62 in *Ppt1* ko retinas (Fig. 6C).

### Thinning of the *Ppt1* ko retina

**Figure 7:**
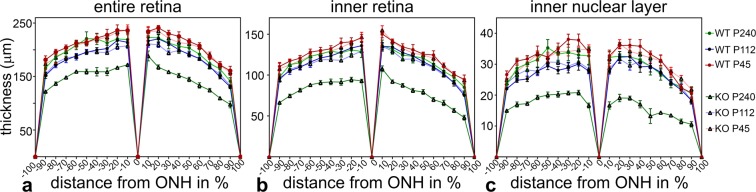
Thickness of the entire retina, inner retina and inner nuclear layer of Ppt1 ko and age-matched wild-type mice. The thickness of the entire retina (**a**), inner retina (**b**) and inner nuclear layer (**c**) was similar in 45- or 112-day-old *Ppt1* ko and wild-type mice, but significantly reduced in P240 mutants when compared to age-matched wild-type mice (p < 0.001 for all comparisons between 240-day-old mutant and wild-type mice; three-way ANOVA followed by a Bonferroni post-hoc test). Values represent the mean ± SEM from six animals of each age and genotype. ONH: optic nerve head.

To analyze the progression and extent of retinal degeneration in *Ppt1* ko mice, we first determined the thickness of the entire retina, the inner retina (i.e. from outer plexiform layer to vitreal retina margin) and the inner nuclear layer in 45-, 112- and 240-day-old mutant and wild-type retinas (Fig. 7). Analyses revealed an age-dependent decrease of the thickness of the entire retina, inner retina and inner nuclear layer of *Ppt1* ko mice when compared to wild-type mice that was independent of the retinal position (effect of the interaction between age and genotype: p < 0.001 according to the three-way ANOVA). Bonferroni post-hoc analyses revealed significant differences between mutant and wild-type retinas only at P240 (entire retina: *Ppt1* ko 147.7 ± 5.5 µm (mean ± SEM), wild-type 199.5 ± 5.7 µm, p < 0.001; inner retina: *Ppt1* ko 81.1 ± 3.6 µm, wild-type 118.1 ± 3.3 µm, p < 0.001; inner nuclear layer: *Ppt1* ko 16.7 ± 0.3 µm, wild-type 29.6 ± 1.0 µm, p < 0.001; Fig. 7a–c). Significant thinning of the entire retina, inner retina and inner nuclear layer at P240 suggests the loss of various retinal cell types in the mutant at advanced stages of the disease.

### Degeneration of photoreceptor cells

**Figure 8:**
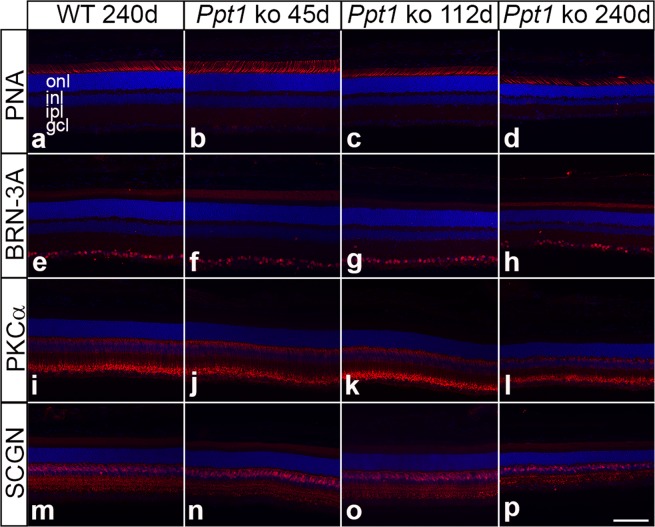
Degeneration of various retinal cell types in PPT1-deficient mice. The density of PNA-labelled cone photoreceptor cells, BRN-3A-positive retinal ganglion cells, PKCα-positive rod bipolar cells and SCGN-positive cone bipolar cells in 45-day-old *Ppt1* ko retinas (**b,f,j,n**, respectively) was similar to that observed in wild-type retinas (**a,e,i,m**, respectively). Retinas from 112-day-old *Ppt1* ko mice also contained normal numbers of retinal ganglion cells (**g**) and rod bipolar cells (**k**), but decreased densities of cone photoreceptor cells (**c**) and cone bipolar cells (**o**). Note the significant decrease in the number of all cell types in 240-day-old mutant retinas (**d,h,l,p**) when compared to age-matched wild-type retinas (a,e,i,m). gcl: ganglion cell layer; inl: inner nuclear layer; ipl: inner plexiform layer; onl: outer nuclear layer. Scale bar in (**p**) for (**a–p**): 100 µm.

**Figure 9:**
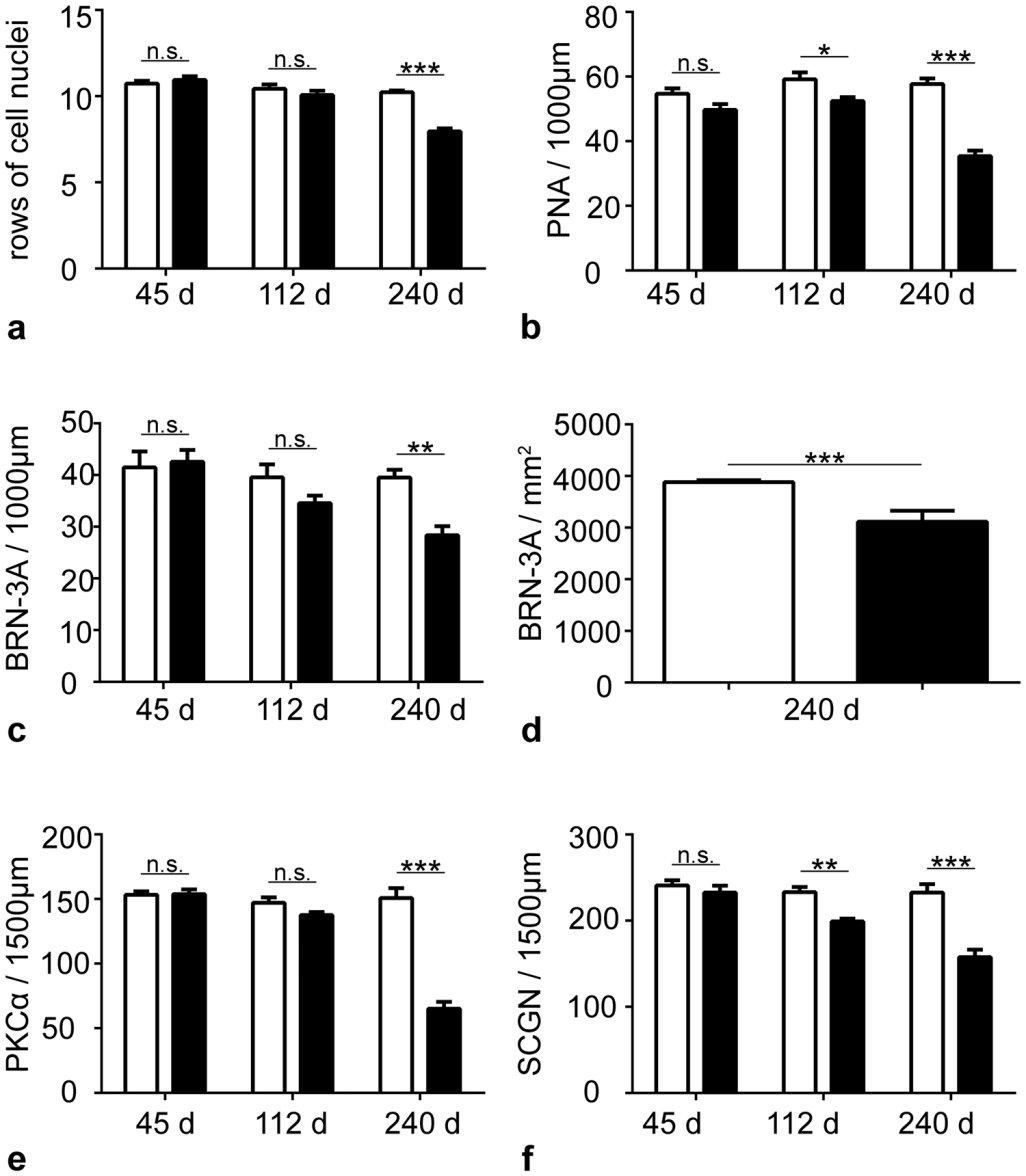
Degeneration of various retinal cell types in Ppt1 ko mice. The number of rows of photoreceptor nuclei (**a**) and the density of PNA-labeled cone photoreceptor cells (**b**), BRN-3A-positive retinal ganglion cells in retinal sections (**c**) or retinal flatmounts (**d**), PKCα-positive rod bipolar cells (**e**) and SCGN-positive cone bipolar cells (**f**) were determined in 45-, 112- and 240-day-old *Ppt1* ko (filled bars) and wild-type mice (open bars). Each bar represents the mean value ± SEM of six animals. Statistical analyses of data were performed with the two-way ANOVA followed by a Bonferroni post-hoc test (**a–c,e,f**) or the Mann-Whitney U test (**d**). n.s.: not significant; *p < 0.05; **p < 0.01; ***p < 0.001.

To analyze the impact of PPT1 deficiency on photoreceptor cells, we first determined the number of rows of DAPI-labelled photoreceptor cell nuclei (Fig. 8). This analysis revealed similar numbers of photoreceptor cells in *Ppt1* ko and age-matched control retinas at P45 and P112 (Fig. 9a). In 240-day-old animals, in comparison, we found 10.2 ± 0.1 (mean ± SEM) rows of photoreceptor cell nuclei in wild-type mice compared to 8.0 ± 0.2 rows of nuclei in mutant mice (p < 0.001; two-way ANOVA followed by a Bonferroni post-hoc test), demonstrating that more than 20% of photoreceptors were lost at this age in the mutant (compare Figs 8a,d, 9a). To study whether cone and rod photoreceptor cells are differentially affected, we next determined the density of PNA-labeled cones (Fig. 8a–d). In 45-day-old animals, the number of cones per 1000 µm retina length was not significantly different between *Ppt1* ko (49.7 ± 1.8; mean ± SEM) and age-matched wild-type mice (54.7 ± 1.6; Fig. 9b). At P112 and P240, however, the number of cones was significantly reduced in mutant retinas by 11.4% (p < 0.05) and 38.7% (p < 0.001) respectively when compared to control retinas (Figs 8a–d, 9b). Together with the progressive loss of photoreceptor cell nuclei, data demonstrate degeneration of both cone and rod photoreceptors, and indicate that cones are more susceptible to PPT1 dysfunction than rods.

### Degeneration of retinal ganglion cells

To study the consequences of PPT1 deficiency on nerve cell types of the inner retina, we next determined the density of BRN-3A-positive ganglion cells. Analyses of retinal sections (Fig. 8e–h) revealed similar densities of ganglion cells in mutant and wild-type retinas at P45 and P112 (Fig. 9c), in line with a normal thickness of the inner retina of *Ppt1* ko mice at these ages (Fig. 7b). At P240, however, the number of ganglion cells per 1000 µm retina length was significantly reduced in the mutant mice (28.4 ± 1.75; mean ± SEM; Fig. 8h) when compared to wild-type mice (39.5 ± 1.5; p < 0.01; two-way ANOVA followed by a Bonferroni post-hoc test; Figs 8e, 9c). A significant loss of ganglion cells in 240-day-old *Ppt1* ko mice was confirmed by analyses of BRN-3A-stained retinal flatmounts (Supplementary Fig. 4) which revealed 3112.8 ± 87.5 ganglion cells/mm^2^ in *Ppt1* ko retinas as opposed to 3879.7 ± 15.5 ganglion cells/mm^2^ in wild-type retinas (Fig. 9d; p < 0.001; Mann-Whitney U test).

### Early and pronounced loss of bipolar cells

Significant thinning of the inner nuclear layer of mutant retinas at P240 (Fig. 7c) suggests the loss of retinal interneurons at late stages of the disease. We therefore immunostained mutant and wild-type retinas with antibodies to PKCα (Fig. 8i–l) and SCGN (Fig. 8m–p) to visualize rod and cone bipolar cells, respectively. Mutant and wild-type retinas contained similar numbers of both bipolar cell types at P45 (Fig. 9e,f). Similarly, there was no significant difference in the density of rod bipolar cells between both genotypes at P112 (Fig. 9e). However, the density of cone bipolar cells was significantly reduced at this age with 199.2 ± 3.1 (mean ± SEM) cone bipolar cells per 1500 µm retina length in *Ppt1* ko retinas compared to 233.2 ± 6.1 cone bipolar cells per 1500 µm retina length in wild-type retinas (Fig. 9f; p < 0.01; two-way ANOVA followed by a Bonferroni post-hoc test). In 240-day-old *Ppt1* ko retinas, we found a pronounced loss of both rod and cone bipolar cells (compare Fig. 8l with i and Fig. 8p with m, respectively). The density of PKCα-positive rod bipolar cells was reduced by 57% from 150.8 ± 7.5 cells per 1500 µm retina length in wild-type retinas to 65.2 ± 5.2 cells per 1500 µm retina length in *Ppt1* ko retinas (Fig. 9e; p < 0.001), while the density of SCGN-positive cone bipolar cells was reduced by 32% from 232.7 ± 9.6 cells per 1500 µm retina length in wild-type retinas to 157.7 ± 8.7 cells per 1500 µm retina length in *Ppt1* ko retinas (Fig. 9f; p < 0.001).

## Discussion

Retinal degeneration and loss of vision is a characteristic clinical symptom of most NCLs, and among the first clinical signs in CLN1 patients^6,9,17,20,43^. We therefore performed an in-depth analysis of the retinal phenotype of a PPT1-deficient mouse^30^ to gain insight into the molecular and cellular changes and the progression of the retinal dystrophy caused by PPT1 dysfunction. Accumulation of storage material, reactive astrogliosis and microgliosis, dysregulation of lysosomal proteins and elevated levels of the autophagy marker SQSTM1/p62 were evident early in the disease course. Progressive degeneration of various retinal cell types, in comparison, became apparent at significantly later stages of the disease.

Accumulation of autofluorescent storage material with a protein composition and ultrastructural appearance characteristic for different NCL forms is a hallmark of the disease. While subunit c of mitochondrial ATP synthase prevails in most NCLs, saposin A and D comprise the major protein components of storage material in CLN1, CLN4 and CLN10 disease^43–46^. In the *Ppt1* ko retina, levels of saposin D were elevated at P45, more than two months before we observed a moderate but significant loss of some retinal cell types. Levels of saposin D further increased until the end stage of the disease when storage material resembling the ultrastructure of granular osmiophilic deposits was found in all retinal cell types and microglia/macrophages. While accumulation of storage material clearly preceding the onset of neurological symptoms has also been noted in other studies, both in CLN1 animal models and CLN1 patients^30,31,44,47^, its relevance for the pathogenesis of the disease is largely unknown. In brains of a sheep CLN6 model, for instance, distribution and amount of storage material correlated only partially with the extent of neurodegeneration^48,49^, and experimental work on *Ctsd* ko mice indicated that nerve cells can tolerate large amounts of storage material^50^.

Reactive astrogliosis and first signs of microglia activation in *Ppt1* ko retinas were also evident more than two months before we observed a significant loss of some retinal cell types. Activation of astrocytes and microglia and infiltration of lymphocytes prior to significant neurodegeneration in CLN1 mouse models suggests a critical role of neuroinflammation in the pathogenesis of the disease^31,35,51–53^. In support of this view, genetic and pharmacological immunomodulatory therapeutic approaches targeting the innate or adaptive immune system have indeed been shown to delay the thinning of inner retinal layers and the loss of retinal ganglion cells in the *Ppt1* ko retina^35,54,55^. Whether the degeneration of other retinal cell types affected in the PPT1-deficient retina was also attenuated by these treatments was, however, not analysed in these studies. Early-onset microgliosis has recently also been reported to closely accompany light-induced retinal degeneration in the *Cln3*^*Δex7/8*^ mouse model of CLN3 disease^56^ and the progressive loss of photoreceptor cells in the *nclf* mouse model of CLN6 disease^57^. Interestingly, treatment of *Cln3*^*Δex7/8*^ mice with the immunomodulatory compound minocycline or of *nclf* mice with the immunomodulatory compounds curcumin or docosahexaenoic acid resulted in attenuation of microgliosis and partial correction of the retina pathology^56,57^.

A slight upregulation of LAMP1, LAMP2, CTSZ and CTSD was another early pathological feature of mutant retinas. Levels of all four lysosomal proteins progressively increased until the end stage of the disease at P240. In line with the immunohistochemical data, immunoblot analyses of retinas at P240 revealed that levels of LAMP1, LAMP2 and CTSZ were increased 1.3-, 2.6- and 2.8-fold, respectively when compared with control retinas. For the aspartic protease CTSD, we found a 2.1-fold increase for the precursor form and a 3.9-fold increase for the mature form. Significantly elevated levels of CTSD mRNA and CTSD protein were also observed in the brain of *Ppt1* ko mice prior to the onset of neurological impairment^58^. Paradoxically, however, levels of the 31 kDa and 14 kDa CTSD fragments, which constitute enzymatically active CTSD after dimerization, were markedly decreased in PPT1-deficient lysosomes^58^. Impaired processing of CTSD in lysosomes correlated with an elevated pH and decreased levels of cathepsin B and cathepsin L. Evidence was presented that the increased levels of mature CTSD detected in immunoblots of total tissue brain homogenates comprised CTSD that was released from the cells and subsequently processed by a matrix metalloproteinase. Of note, extracellularly located CTSD was shown to be neurotoxic and might thus contribute to the pathogenesis of CLN1 disease, in addition to the paucity of CTSD enzymatic activity in PPT1-deficient lysosomes^58^.

Upregulation of lysosomal enzyme expression in response to transcription factor EB (TFEB) activation represents an adaptive mechanism in conditions of lysosomal dysfunction and starvation^59^. Under normal conditions, the lysosome-associated mammalian target of rapamycin complex 1 (mTORC1) phosphorylates TFEB, thereby retaining the transcription factor in the cytosol in an inactive state^60^. Upregulation of TFEB mRNA and protein, primarily in astrocytes and microglia, has been observed in the brain of CLN1-deficient mice^58^, and pharmacological inhibition of PPT1 activity by DQ661 has been shown to repress mTORC1 activity and to block autophagic flux^61^. Together, results implicate TFEB in both the elevated expression levels of lysosomal enzymes and the blockage of autophagic flux observed in the PPT1-deficient retina. We have recently found elevated levels of several lysosomal proteins also in retinas of a CLN6^37^ and a CLN7 mouse model^38^. While the relevance of the marked upregulation of lysosomal protein biosynthesis for the progression of the retinal dystrophies is unknown, it might serve as a useful pathogenic marker for studies aimed at correcting lysosomal dysfunction in the *Ppt1* ko retina as we have recently demonstrated in *Ctsd* ko mice after intravitreal injections of human pro-CTSD^62^.

At the morphological level, PPT1 dysfunction has been reported to result in a slight thinning of the outer nuclear layer and a moderate loss of photoreceptor cells late during the disease course^31,32,34,36^. In line with these findings, we found normal photoreceptor numbers in 112-day-old *Ppt1* ko mice, but significantly reduced photoreceptor numbers in 240-day-old mutants. Analyses specifically of cones, which comprise only ~3% of all photoreceptors in the mouse retina^63^ revealed, however, that photoreceptor degeneration becomes evident already at P112, as indicated by a ~11% loss of cones. At P240, cones were markedly reduced by ~39%. In comparison and in agreement with other studies^34,36^, the number of all photoreceptor cells (i.e. cones and rods) at the end stage of the disease was decreased by ~20%, demonstrating that cones are more severely affected than rods. However, a significant decline in scotopic and photopic ERG amplitudes has been observed before the onset of photoreceptor loss in 2- and 3-month-old mutants, respectively. Similarly, another study found moderately decreased ERG responses in 4-month-old *Ppt1* ko mice^36^. Early deterioration of retina function but a relatively late and moderate photoreceptor loss indicates that PPT1 dysfunction impairs photoreceptor function in addition to photoreceptor survival. Notably, it has recently been shown that PPT1 is localized in cilia, and that PPT1 deficiency affected the abundance levels and distribution of ciliary proteins in presymptomatic *Ppt1* ko mice^64^. Furthermore, PPT1 deficiency resulted in a reduced number of ciliated cells and in the formation of abnormally long cilia. Together these data suggest that cilia abnormalities, the cause of several isolated and syndromic retinal dystrophies^65–67^, might contribute to the pathogenesis of CLN1 disease^64^.

Retinal ganglion cells comprise another cell type affected by PPT1 dysfunction^32,35^. Using antibodies to BRN-3A, an established marker to monitor ganglion cell degeneration^68^, we found normal RGC numbers in 112-day-old mutants. At P240, in comparison, analyses of retinal sections and flatmount preparations revealed a loss of RGCs by ~28% and ~20%. respectively. Another study has reported an earlier onset and more pronounced loss of RGCs^35^. The reasons for these discrepant findings are unknown given that the same CLN1 mouse model on the same genetic background was analyzed with similar methods in both studies.

PPT1 dysfunction additionally results in thinning of the inner nuclear layer^32,36^. One study has reported normal cell numbers in this layer in four-month-old animals, and a cell loss of ~26% in eight-month-old mutants^36^. However, no attempts were made to identify the affected cell types and to study the time course of their degeneration. Our results demonstrate a significant loss of SCGN-positive cone bipolar cells but normal numbers of PKCα-positive rod bipolar cells at P112. At the end stage of the disease at P240, numbers of both cone and rod bipolar cells were markedly reduced by 32% and 57%, respectively. Of note, elevated levels of SQSTM1/p62, a cargo-receptor that binds ubiquitin and LC3 and is required for autophagic degradation^69^, were restricted to the inner nuclear layer long before the onset and during the entire course of the retinal dystrophy. Furthermore, we observed an only minor co-localization of SQSTM1/p62-positive aggregates with the lysosomal marker protein LAMP1, indicating mainly extralysosomal accumulation of SQSTM1/p62. Extralysosomal accumulation of SQSTM1/p62-positive aggregates has also been observed in neurons of *Cln2* ko mice^70^ and arylsulfatase G-deficient mice^71^, most likely as a result of lysosomal membrane permeability. Alternatively, accumulation of lysosomal storage material may impair the fusion of autophagosomes with lysosomes and thus the processing of autophagic material, resulting in accumulation of SQSTM1/p62-positive aggregates^72^. Increased levels of the autophagic markers SQSTM1/p62 and/or microtubule-associated protein 1 light chain 3-II (LC3-II) as pathological markers for impaired constitutive autophagy have also been reported in mouse models of various other NCLs, both in the brain^41,73–75^ and in the retina^62,76,77^.

The pronounced loss of bipolar cells is in line with the decreased b/a-amplitude ratio observed in ERG recordings from CLN1 mouse models^32,36^. Photopic and scotopic ERG recordings from CLN1 patients at relatively early stages of the disease also revealed a pronounced reduction of the b-wave amplitude with a relative preservation of the a-wave amplitude^17^. Interestingly, an involvement of the inner nuclear layer early during the disease course has also been demonstrated for other NCLs. For instance, a progressive decrease of the b/a amplitude ratio and/or a pronounced degeneration of inner nuclear layer neurons has been observed in a canine CLN2 model and CLN2 patients, and in a CLN3 mouse model and CLN3 patients^16,78–82^. Of interest in this context, a recent study has provided evidence that the progressive loss of photoreceptor cells in the *nclf* mouse model of CLN6 disease^37,57^ is caused by dysfunctions of bipolar cells^83^. While an AAV vector-mediated expression of *CLN6* in photoreceptor cells had no beneficial effects on photoreceptor survival or function, photoreceptor cells were preserved and deterioration of retinal function was attenuated when CLN6 was expressed in bipolar cells, which in the *nclf* mouse start to degenerate only at late stages of the disease^83^. The combined data suggest that retinal interneurons are particularly vulnerable to lysosomal dysfunctions, at least in certain NCLs.

Enzyme replacement strategies through injection of the recombinant proteins, AAV vector-mediated gene transfer or cell transplantation represent a promising treatment strategy for NCLs caused by dysfunctions of soluble lysosomal enzymes (for reviews, see^6,7,84–88^). In support of this view, a phase I/II clinical trial has recently demonstrated that biweekly intracerebroventricular infusions of a recombinant human tripeptidyl peptidase 1 (TPP1) proenzyme (cerliponase alfa) resulted in significant attenuation of disease progression in CLN2 patients^89^. Preclinical work suggests, however, that retinal degeneration in these patients will likely proceed at a normal rate despite the successful brain-directed treatment. For instance, intraventricular injections of recombinant TPP1 or TPP1-encoding AAV vectors significantly delayed onset and progression of the brain pathology in a CLN2 canine model^90,91^, but had no therapeutic impact on the progression of retinal degeneration and visual deterioration^92^. Similarly, injections of a CTSD-encoding AAV vector into the cerebral hemispheres of neonatal *Ctsd* ko mice resulted in significant attenuation of neurodegeneration in the brain but not in the retina^50^. While the combined data indicate the urgent need to develop enzyme replacement strategies that specifically target the retina, only a few studies have analyzed the efficacy of this approach to treat retinal dystrophies^34,62,93^. In the *Ppt1* ko mouse, intravitreal injections of an AAV2 vector encoding human PPT1 have been shown to partly preserve retina function^34^. However, loss of photoreceptor cells was not prevented despite the fact that levels of PPT1 enzyme activity in treated retinas exceeded by far those found in healthy control retinas. While the reasons for the limited effect on photoreceptor survival are unknown, separate analyses of rod and cone survival were not performed, and potential therapeutic effects on the other cell types affected in the *Ppt1* ko retina were not evaluated^34^.

In summary, our data demonstrate early pathological alterations in the *Ppt1* ko retina, and a progressive loss of various retinal cell types at relatively late stages of the disease. The detailed knowledge of the progression of the retinal dystrophy at the cellular and molecular level will aid future work aimed at developing treatments for vision loss in CLN1 disease.

## Supplementary information

Supplementary information

## Data Availability

The datasets generated and/or analysed during the current study are available from the corresponding author on request.

## References

[1] Haltia M (2006). The neuronal ceroid-lipofuscinoses: from past to present. Biochimica et biophysica acta.

[2] Haltia M, Goebel HH (2013). The neuronal ceroid-lipofuscinoses: a historical introduction. Biochimica et biophysica acta.

[3] Williams, R. E. In *The Neuronal Ceroid Lipofuscinosis (Batten Disease)*. (eds Mole, S. E., Williams, R. E. & Goebel, H. H.) 361–365 (Oxford University Press, 2011).

[4] Williams RE, Mole SE (2012). New nomenclature and classification scheme for the neuronal ceroid lipofuscinoses. Neurology.

[5] Kollmann K (2013). Cell biology and function of neuronal ceroid lipofuscinosis-related proteins. Biochimica et biophysica acta.

[6] Kohlschütter A, Schulz A, Bartsch U, Storch S (2019). Current and emerging treatment strategies for neuronal ceroid lipofuscinoses. CNS drugs.

[7] Johnson TB (2019). Therapeutic landscape for Batten disease: current treatments and future prospects. Nature reviews. Neurology.

[8] Carcel-Trullols J, Kovacs AD, Pearce DA (2015). Cell biology of the NCL proteins: What they do and don’t do. Biochimica et biophysica acta.

[9] Schulz A, Kohlschütter A, Mink J, Simonati A, Williams R (2013). NCL diseases - clinical perspectives. Biochimica et biophysica acta.

[10] Sleat DE, Gedvilaite E, Zhang Y, Lobel P, Xing J (2016). Analysis of large-scale whole exome sequencing data to determine the prevalence of genetically-distinct forms of neuronal ceroid lipofuscinosis. Gene.

[11] Santavuori P, Haltia M, Rapola J, Raitta C (1973). Infantile type of so-called neuronal ceroid-lipofuscinosis. 1. A clinical study of 15 patients. J Neurol Sci.

[12] Goebel HH (1995). The neuronal ceroid-lipofuscinoses. Journal of child neurology.

[13] Vesa J (1995). Mutations in the palmitoyl protein thioesterase gene causing infantile neuronal ceroid lipofuscinosis. Nature.

[14] Chattopadhyay S, Pearce DA (2000). Neural and extraneural expression of the neuronal ceroid lipofuscinoses genes CLN1, CLN2, and CLN3: functional implications for CLN3. Molecular genetics and metabolism.

[15] Dearborn JT (2016). Histochemical localization of palmitoyl protein thioesterase-1 activity. Molecular genetics and metabolism.

[16] Weleber RG (1998). The dystrophic retina in multisystem disorders: the electroretinogram in neuronal ceroid lipofuscinoses. Eye.

[17] Weleber RG (2004). Electroretinographic and clinicopathologic correlations of retinal dysfunction in infantile neuronal ceroid lipofuscinosis (infantile Batten disease). Molecular genetics and metabolism.

[18] Jalanko A, Braulke T (2009). Neuronal ceroid lipofuscinoses. Biochimica et biophysica acta.

[19] Santavuori P (1988). Neuronal ceroid-lipofuscinoses in childhood. Brain & development.

[20] Mole SE, Williams RE, Goebel HH (2005). Correlations between genotype, ultrastructural morphology and clinical phenotype in the neuronal ceroid lipofuscinoses. Neurogenetics.

[21] Chabrol B, Caillaud C, Minassian B (2013). Neuronal ceroid lipofuscinoses. Handbook of clinical neurology.

[22] Das AK (1998). Molecular genetics of palmitoyl-protein thioesterase deficiency in the U.S. The Journal of clinical investigation.

[23] Kalviainen R (2007). Juvenile-onset neuronal ceroid lipofuscinosis with infantile CLN1 mutation and palmitoyl-protein thioesterase deficiency. Eur J Neurol.

[24] Mole, S. E., Mitchison, H. M. & Munroe, P. B. Molecular basis of the neuronal ceroid lipofuscinoses: mutations in CLN1, CLN2, CLN3, and CLN5. *Human mutation* **14**, 199–215, doi:10.1002/(SICI)1098-1004(1999)14:3<199::AID-HUMU3>3.0.CO;2-A (1999). 10.1002/(SICI)1098-1004(1999)14:3<199::AID-HUMU3>3.0.CO;2-A 10477428

[25] Ramadan H (2007). Adult neuronal ceroid lipofuscinosis caused by deficiency in palmitoyl protein thioesterase 1. Neurology.

[26] van Diggelen OP (2001). Adult neuronal ceroid lipofuscinosis with palmitoyl-protein thioesterase deficiency: first adult-onset patients of a childhood disease. Annals of neurology.

[27] Mazzei R (2002). A novel mutation in the CLN1 gene in a patient with juvenile neuronal ceroid lipofuscinosis. Journal of neurology.

[28] Metelitsina TI, Waggoner DJ, Grassi MA (2016). Batten disease caused by a novel mutation in the *PPT1*Gene. Retin Cases Brief Rep.

[29] Birch DG (1999). Retinal degeneration in retinitis pigmentosa and neuronal ceroid lipofuscinosis: An overview. Molecular genetics and metabolism.

[30] Gupta P (2001). Disruption of PPT1 or PPT2 causes neuronal ceroid lipofuscinosis in knockout mice. Proceedings of the National Academy of Sciences of the United States of America.

[31] Jalanko A (2005). Mice with Ppt1Deltaex4 mutation replicate the INCL phenotype and show an inflammation-associated loss of interneurons. Neurobiology of disease.

[32] Bouchelion A, Zhang Z, Li Y, Qian H, Mukherjee AB (2014). Mice homozygous for c.451C>T mutation in Cln1 gene recapitulate INCL phenotype. Annals of clinical and translational neurology.

[33] Miller JN, Kovacs AD, Pearce DA (2015). The novel Cln1(R151X) mouse model of infantile neuronal ceroid lipofuscinosis (INCL) for testing nonsense suppression therapy. Human molecular genetics.

[34] Griffey M, Macauley SL, Ogilvie JM, Sands MS (2005). AAV2-mediated ocular gene therapy for infantile neuronal ceroid lipofuscinosis. Molecular therapy: the journal of the American Society of Gene Therapy.

[35] Groh J (2013). Immune cells perturb axons and impair neuronal survival in a mouse model of infantile neuronal ceroid lipofuscinosis. Brain: a journal of neurology.

[36] Lei B, Tullis GE, Kirk MD, Zhang K, Katz ML (2006). Ocular phenotype in a mouse gene knockout model for infantile neuronal ceroid lipofuscinosis. Journal of neuroscience research.

[37] Bartsch U (2013). Apoptotic photoreceptor loss and altered expression of lysosomal proteins in the nclf mouse model of neuronal ceroid lipofuscinosis. Investigative ophthalmology & visual science.

[38] Jankowiak W (2016). Retinal degeneration in mice deficient in the lysosomal membrane protein CLN7. Investigative ophthalmology & visual science.

[39] Kruszewski K, Lüllmann-Rauch R, Dierks T, Bartsch U, Damme M (2016). Degeneration of photoreceptor cells in arylsulfatase G-deficient mice. Investigative ophthalmology & visual science.

[40] Flachsbarth K (2014). Neural stem cell-based intraocular administration of ciliary neurotrophic factor attenuates the loss of axotomized ganglion cells in adult mice. Investigative ophthalmology & visual science.

[41] Brandenstein L, Schweizer M, Sedlacik J, Fiehler J, Storch S (2016). Lysosomal dysfunction and impaired autophagy in a novel mouse model deficient for the lysosomal membrane protein Cln7. Human molecular genetics.

[42] Palmer DN, Barry LA, Tyynelä J, Cooper JD (2013). NCL disease mechanisms. Biochimica et biophysica acta.

[43] Radke J, Stenzel W, Goebel HH (2015). Human NCL Neuropathology. Biochimica et biophysica acta.

[44] Anderson GW, Goebel HH, Simonati A (2013). Human pathology in NCL. Biochimica et biophysica acta.

[45] Tyynelä J, Palmer DN, Baumann M, Haltia M (1993). Storage of saposins A and D in infantile neuronal ceroid-lipofuscinosis. FEBS letters.

[46] Palmer DN (2015). The relevance of the storage of subunit c of ATP synthase in different forms and models of Batten disease (NCLs). Biochimica et biophysica acta.

[47] Galvin N (2008). A murine model of infantile neuronal ceroid lipofuscinosis-ultrastructural evaluation of storage in the central nervous system and viscera. Pediatr Dev Pathol.

[48] Oswald MJ (2005). Glial activation spreads from specific cerebral foci and precedes neurodegeneration in presymptomatic ovine neuronal ceroid lipofuscinosis (CLN6). Neurobiology of disease.

[49] Kay GW, Jay NP, Palmer DN (2011). The specific loss of GnRH-positive neurons from the hypothalamus of sheep with CLN6 neuronal ceroid lipofuscinosis occurs without glial activation and has only minor effects on reproduction. Neurobiology of disease.

[50] Shevtsova Z (2010). CNS-expressed cathepsin D prevents lymphopenia in a murine model of congenital neuronal ceroid lipofuscinosis. The American journal of pathology.

[51] Kielar C (2007). Successive neuron loss in the thalamus and cortex in a mouse model of infantile neuronal ceroid lipofuscinosis. Neurobiology of disease.

[52] Macauley SL (2014). An anti-neuroinflammatory that targets dysregulated glia enhances the efficacy of CNS-directed gene therapy in murine infantile neuronal ceroid lipofuscinosis. The Journal of neuroscience: the official journal of the Society for Neuroscience.

[53] Macauley SL, Pekny M, Sands MS (2011). The role of attenuated astrocyte activation in infantile neuronal ceroid lipofuscinosis. The Journal of neuroscience: the official journal of the Society for Neuroscience.

[54] Groh J (2016). Sialoadhesin promotes neuroinflammation-related disease progression in two mouse models of CLN disease. Glia.

[55] Groh J, Berve K, Martini R (2017). Fingolimod and Teriflunomide Attenuate Neurodegeneration in Mouse Models of Neuronal Ceroid Lipofuscinosis. Molecular therapy: the journal of the American Society of Gene Therapy.

[56] Dannhausen Katharina, Möhle Christoph, Langmann Thomas (2018). Immunomodulation with minocycline rescues retinal degeneration in juvenile neuronal ceroid lipofuscinosis mice highly susceptible to light damage. Disease Models & Mechanisms.

[57] Mirza M (2013). Progressive retinal degeneration and glial activation in the CLN6 (nclf) mouse model of neuronal ceroid lipofuscinosis: a beneficial effect of DHA and curcumin supplementation. PloS one.

[58] Chandra G (2015). Cln1 gene disruption in mice reveals a common pathogenic link between two of the most lethal childhood neurodegenerative lysosomal storage disorders. Human molecular genetics.

[59] Settembre C., Ballabio A. (2014). Lysosomal Adaptation: How the Lysosome Responds to External Cues. Cold Spring Harbor Perspectives in Biology.

[60] Settembre C (2012). A lysosome-to-nucleus signalling mechanism senses and regulates the lysosome via mTOR and TFEB. The EMBO journal.

[61] Nicastri MC, Rebecca VW, Amaravadi RK, Winkler JD (2018). Dimeric quinacrines as chemical tools to identify PPT1, a new regulator of autophagy in cancer cells. Mol Cell Oncol.

[62] Marques, A. R. A. *et al*. Enzyme replacement therapy with recombinant pro-CTSD (cathepsin D) corrects defective proteolysis and autophagy in neuronal ceroid lipofuscinosis. *Autophagy*, 10.1080/15548627.2019.1637200 (2019). 10.1080/15548627.2019.1637200 PMC7158922 31282275

[63] Jeon CJ, Strettoi E, Masland RH (1998). The major cell populations of the mouse retina. The Journal of neuroscience: the official journal of the Society for Neuroscience.

[64] Segal-Salto M (2017). Proteomics insights into infantile neuronal ceroid lipofuscinosis (CLN1) point to the involvement of cilia pathology in the disease. Human molecular genetics.

[65] Bujakowska Kinga M., Liu Qin, Pierce Eric A. (2017). Photoreceptor Cilia and Retinal Ciliopathies. Cold Spring Harbor Perspectives in Biology.

[66] Estrada-Cuzcano A, Roepman R, Cremers FP, den Hollander AI, Mans DA (2012). Non-syndromic retinal ciliopathies: translating gene discovery into therapy. Human molecular genetics.

[67] May-Simera H, Nagel-Wolfrum K, Wolfrum U (2017). Cilia - The sensory antennae in the eye. Progress in retinal and eye research.

[68] Mead B (2014). Comparative evaluation of methods for estimating retinal ganglion cell loss in retinal sections and wholemounts. PloS one.

[69] Pankiv S (2007). p62/SQSTM1 binds directly to Atg8/LC3 to facilitate degradation of ubiquitinated protein aggregates by autophagy. The Journal of biological chemistry.

[70] Micsenyi MC, Sikora J, Stephney G, Dobrenis K, Walkley SU (2013). Lysosomal membrane permeability stimulates protein aggregate formation in neurons of a lysosomal disease. The Journal of neuroscience: the official journal of the Society for Neuroscience.

[71] Kowalewski B (2015). Ataxia is the major neuropathological finding in arylsulfatase G-deficient mice: similarities and dissimilarities to Sanfilippo disease (mucopolysaccharidosis type III). Human molecular genetics.

[72] Settembre C (2008). A block of autophagy in lysosomal storage disorders. Human molecular genetics.

[73] Thelen M (2012). Disruption of the autophagy-lysosome pathway is involved in neuropathology of the nclf mouse model of neuronal ceroid lipofuscinosis. PloS one.

[74] Koike M (2005). Participation of autophagy in storage of lysosomes in neurons from mouse models of neuronal ceroid-lipofuscinoses (Batten disease). The American journal of pathology.

[75] Tanaka Y, Chambers JK, Matsuwaki T, Yamanouchi K, Nishihara M (2014). Possible involvement of lysosomal dysfunction in pathological changes of the brain in aged progranulin-deficient mice. Acta neuropathologica communications.

[76] Leinonen H (2017). Retinal degeneration in a mouse model of CLN5 disease is associated with compromised autophagy. Scientific reports.

[77] von Eisenhart-Rothe P (2018). Failure of autophagy-lysosomal pathways in rod photoreceptors causes the early retinal degeneration phenotype observed in *Cln6*^*nclf*^mice. Investigative ophthalmology & visual science.

[78] Katz ML (2008). Retinal pathology in a canine model of late infantile neuronal ceroid lipofuscinosis. Investigative ophthalmology & visual science.

[79] Katz ML, Johnson GS, Tullis GE, Lei B (2008). Phenotypic characterization of a mouse model of juvenile neuronal ceroid lipofuscinosis. Neurobiology of disease.

[80] Collins J, Holder GE, Herbert H, Adams GG (2006). Batten disease: features to facilitate early diagnosis. The British journal of ophthalmology.

[81] Staropoli JF (2012). Large-scale phenotyping of an accurate genetic mouse model of JNCL identifies novel early pathology outside the central nervous system. PloS one.

[82] Volz C, Mirza M, Langmann T, Jägle H (2014). Retinal function in aging homozygous Cln3 (Deltaex7/8) knock-in mice. Advances in experimental medicine and biology.

[83] Kleine Holthaus SM (2018). Prevention of photoreceptor cell loss in a *Cln6*^*nclf*^ mouse model of batten disease requires *CLN6* gene transfer to bipolar cells. Molecular therapy: the journal of the American Society of Gene Therapy.

[84] Geraets RD (2016). Moving towards effective therapeutic strategies for neuronal ceroid lipofuscinosis. Orphanet journal of rare diseases.

[85] Neverman NJ, Best HL, Hofmann SL, Hughes SM (2015). Experimental therapies in the neuronal ceroid lipofuscinoses. Biochimica et biophysica acta.

[86] Sands MS (2013). Considerations for the treatment of infantile neuronal ceroid lipofuscinosis (infantile Batten disease). Journal of child neurology.

[87] Mole SE (2019). Clinical challenges and future therapeutic approaches for neuronal ceroid lipofuscinosis. The Lancet. Neurology.

[88] Hawkins-Salsbury JA, Cooper JD, Sands MS (2013). Pathogenesis and therapies for infantile neuronal ceroid lipofuscinosis (infantile CLN1 disease). Biochimica et biophysica acta.

[89] Schulz A (2018). Study of intraventricular cerliponase alfa for CLN2 disease. The New England journal of medicine.

[90] Katz ML (2014). Enzyme replacement therapy attenuates disease progression in a canine model of late-infantile neuronal ceroid lipofuscinosis (CLN2 disease). Journal of neuroscience research.

[91] Katz ML (2015). AAV gene transfer delays disease onset in a TPP1-deficient canine model of the late infantile form of Batten disease. Sci Transl Med.

[92] Whiting RE (2016). Intracerebroventricular gene therapy that delays neurological disease progression is associated with selective preservation of retinal ganglion cells in a canine model of CLN2 disease. Experimental eye research.

[93] Tracy CJ (2016). Intravitreal implantation of TPP1-transduced stem cells delays retinal degeneration in canine CLN2 neuronal ceroid lipofuscinosis. Experimental eye research.

[94] Klein A (1994). Sphingolipid activator protein D (sap-D) stimulates the lysosomal degradation of ceramide *in vivo*. Biochemical and biophysical research communications.

