# Supplementary material for: Mice deficient in the lysosomal enzyme palmitoyl-protein thioesterase 1 (PPT1) display a complex retinal phenotype
Source: Sci Rep. 2019 Oct 2;9:14185. doi: 10.1038/s41598-019-50726-8 (PMC6775149; doi:10.1038/s41598-019-50726-8)
Supplement: Supplementary file 1 — Supplementary information [file 41598_2019_50726_MOESM1_ESM.pdf]

Mice deficient in the lysosomal enzyme palmitoyl-protein thioesterase 1 (PPT1) display a complex retinal phenotype

Yevgeniya Atiskova[1], Susanne Bartsch[1], Tatyana Danyukova[2,4], Elke Becker[1], Christian Hagel[3], Stephan Storch[2], Udo Bartsch[1,*]

(1) Department of Ophthalmology, Experimental Ophthalmology (2) Children´s Hospital, Department of Biochemistry and (3) Institute of Neuropathology; University Medical Center Hamburg-Eppendorf, Hamburg, Germany
(4) present address: Department of Osteology and Biomechanics, University Medical Center Hamburg-Eppendorf, Hamburg, Germany

(*) Corresponding author:
Udo Bartsch, PhD
Department of Ophthalmology
University Medical Center Hamburg-Eppendorf
Martinistr. 52
20246 Hamburg, Germany

phone: +49-40-7410-55945
fax: +49-40-7410-55017
email: ubartsch@uke.de

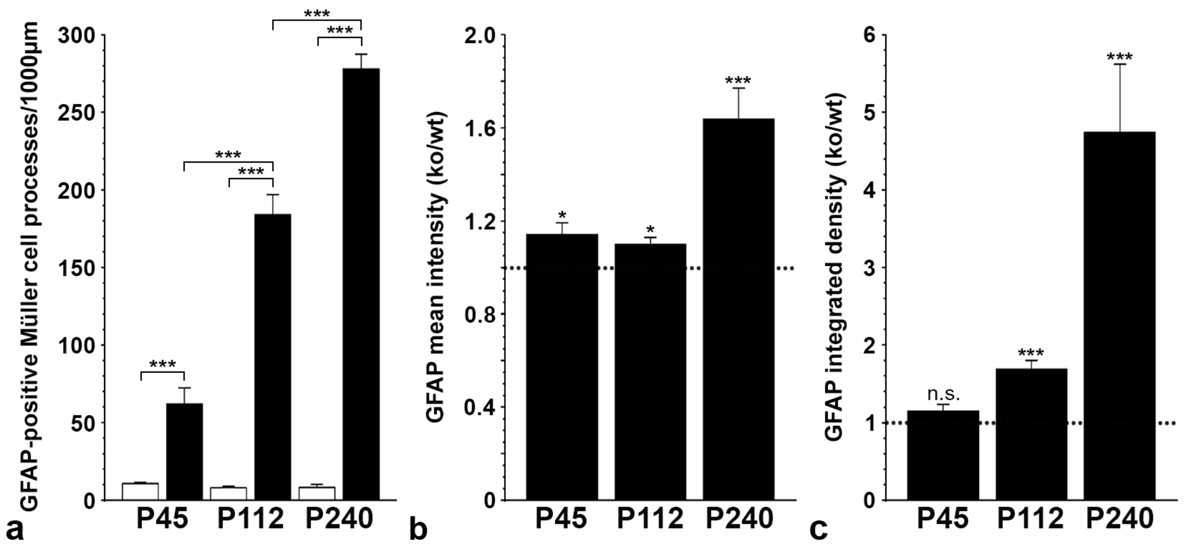

Supplementary Figure 1: Density of GFAP-positive Müller cell processes and expression levels of GFAP in retinal astrocytes.

The number of GFAP-positive Müller cell processes was significantly higher in P45, P112 and P240 *Ppt1* ko retinas (filled bars) than in age-matched wild-type retinas (open bars), and increased significantly in mutant mice with increasing age of the animals (a). Bars in (a) represent mean values ±SEM from six animals for each age and genotype (***: $p<0.001$; two-way ANOVA followed by a Bonferroni post-hoc test). The mean intensity of the GFAP immunostaining signal in retinal astrocytes of mutant retinas was slightly but significantly increased at P45 and P112, and strongly elevated at P240 when compared to age-matched control retinas (b). The integrated density of the GFAP immunostaining signal, in comparison, was not significantly different in retinal astrocytes of *Ppt1* ko and wild-type retinas at P45, but was significantly increased in mutant mice at P112 and P240 when compared to wild-type mice (c). Bars in (b) and (c) represent the quotients (mean ±SEM) of values determined in mutant and wild-type retinas that were processed in parallel in the same immunostainings (n=6 for each genotype and age; *: $p<0.05$; ***: $p<0.001$; paired Student´s t-test). n.s.: not significant.

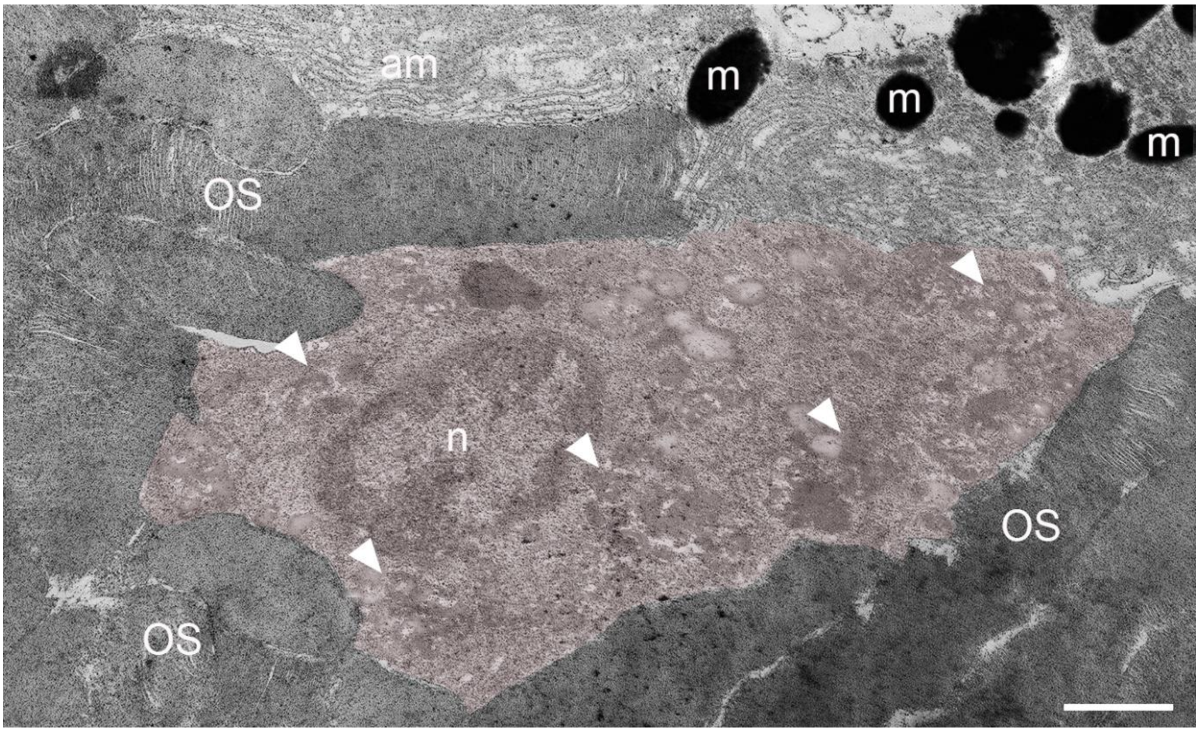

Supplementary Figure 2: *A subretinally located macrophage in a* Ppt1 *ko mouse.*

Electron micrograph of a macrophage (outlined in light red) in a P240 *Ppt1* ko retina located between the outer segments (OS) of photoreceptor cells and retinal pigment epithelial cells. Note that the macrophage is filled with storage material (white arrowheads). am: apical microvilli; m: melanosome; n: nucleus. Scale bar: 1 µm.

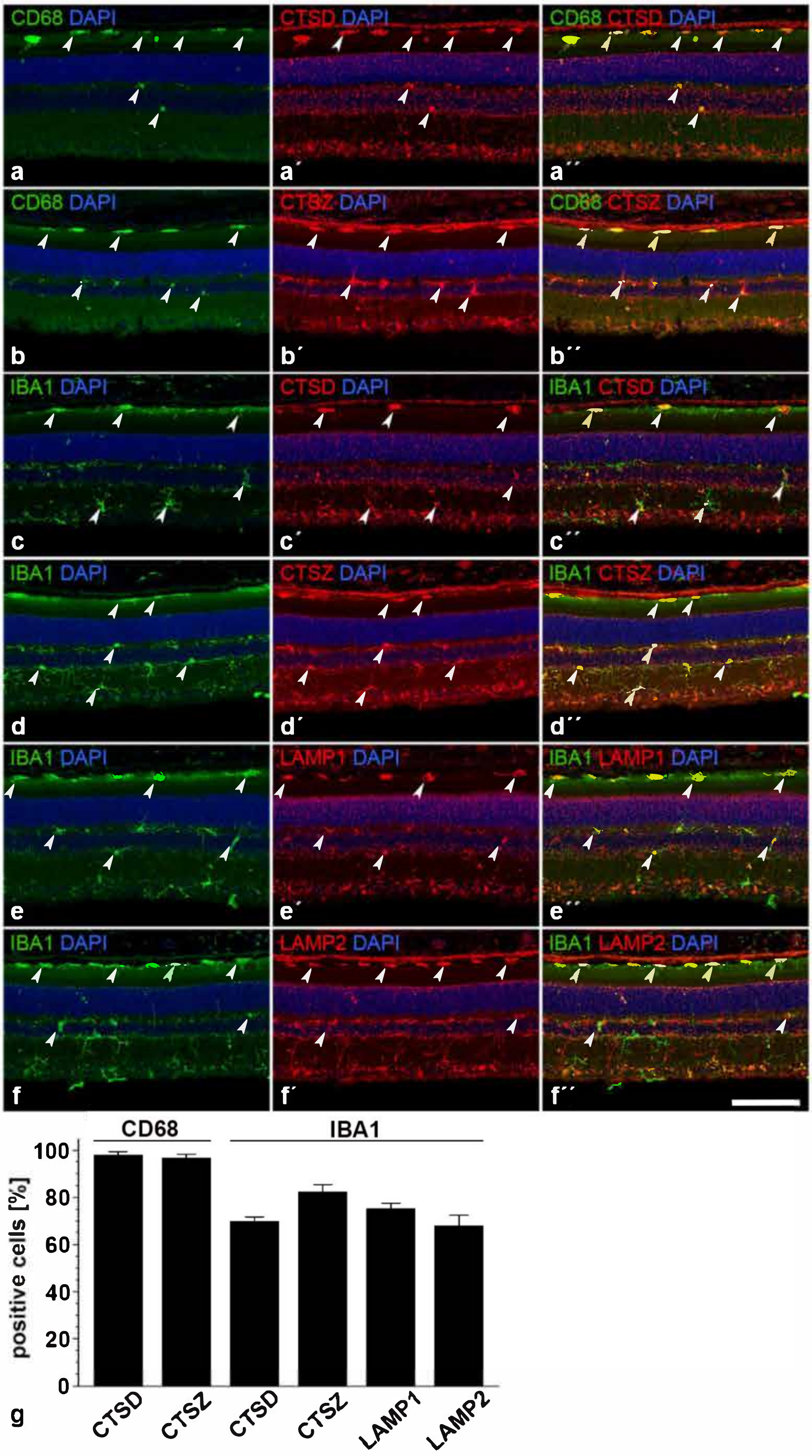

CD68 DAPI
CTSD DAPI
CD68 CTSD DAPI
a
a´
a´´
CD68 DAPI
CTSZ DAPI
CD68 CTSZ DAPI
b
b´
b´´
IBA1 DAPI
CTSD DAPI
IBA1 CTSD DAPI
c
c´
c´´
IBA1 DAPI
CTSZ DAPI
IBA1 CTSZ DAPI
d
d´
d´´
IBA1 DAPI
LAMP1 DAPI
IBA1 LAMP1 DAPI
e
e´
e´´
IBA1 DAPI
LAMP2 DAPI
IBA1 LAMP2 DAPI
f
f´
f´´
CD68
IBA1
positive cells [%]
0
20
40
60
80
100
CTSD
CTSZ
CTSD
CTSZ
LAMP1
LAMP2
g

Supplementary Figure 3: *Elevated levels of lysosomal proteins in CD68- and IBA1-positive microglia/macrophages.*

Double immunostainings of P240 *Ppt1* ko retinas revealed high levels of CTSD- (a-a'') and CTSZ-immunoreactivity (b-b'') in the majority of CD68-positive cells, and of CTSD- (c-c''), CTSZ- (d-d''), LAMP1- (e-e'') and LAMP2- (f-f'') immunoreactivity in the majority of IBA1-positive cells (some marked with white arrowheads in a-f´´). The percentage of CD68- or IBA1-positive cells showing strong CTSD-, CTSZ-, LAMP1- or LAMP2-immunoreactivity (g). Each bar in (g) represents the mean value ±SEM from six animals. Scale bar in (f´´) for (a-f''): 100 µm.

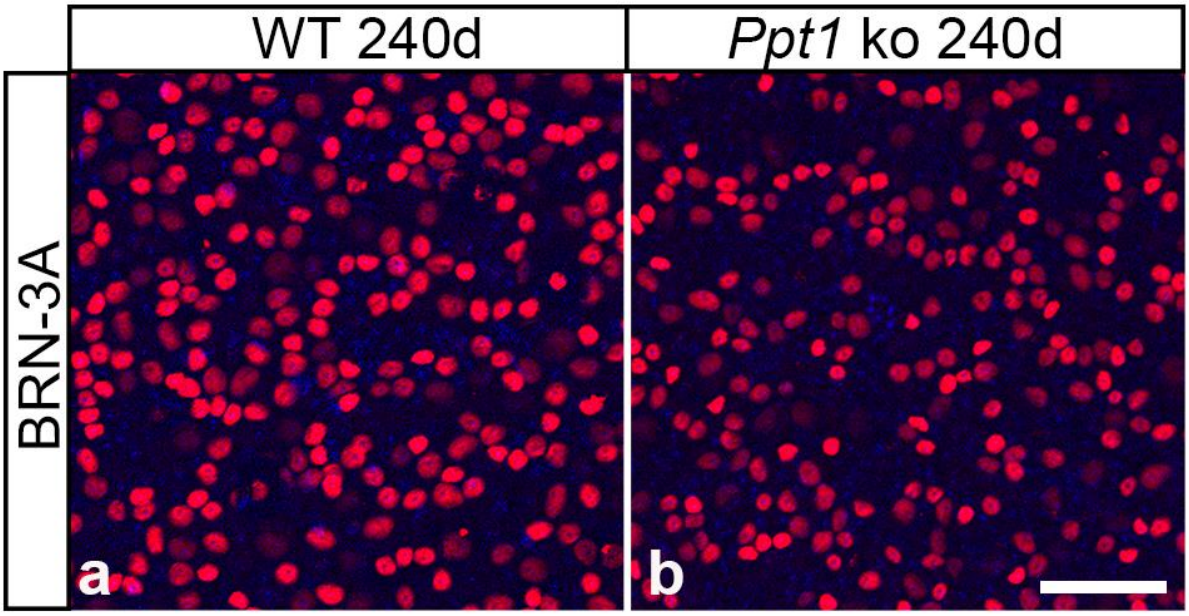

Supplementary Figure 4: *Loss of retinal ganglion cells in* Ppt1 *ko retinas.*

Retinal ganglion cells were visualized in retinal flatmounts from 240-day-old wild-type (a) and *Ppt1* ko mice (b) using anti-BRN-3A antibodies. Note the significantly decreased density of labelled cells in the mutant retina. Scale bar in (b) for (a,b): 50 µm.

(A) Supplemental data for Figure 2

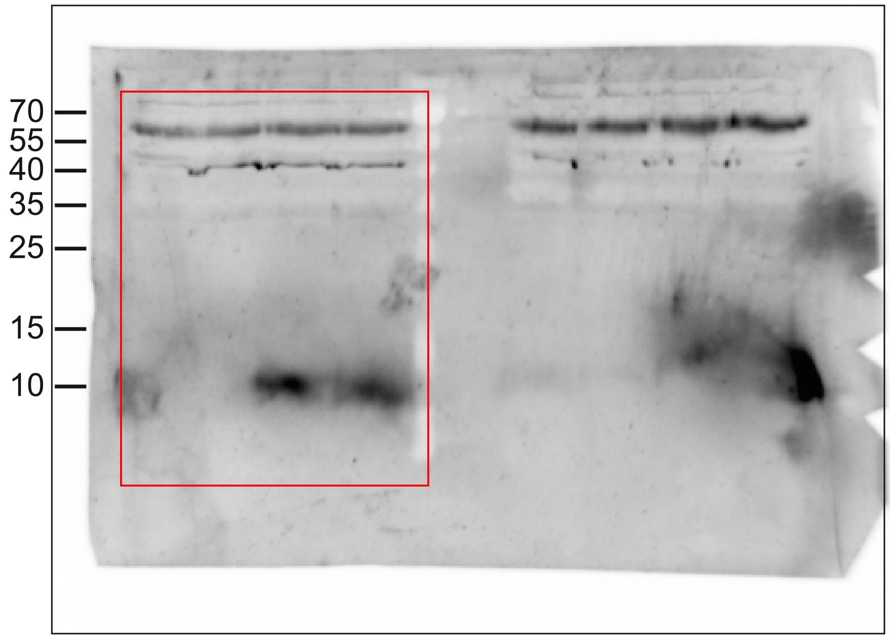

Saposin D

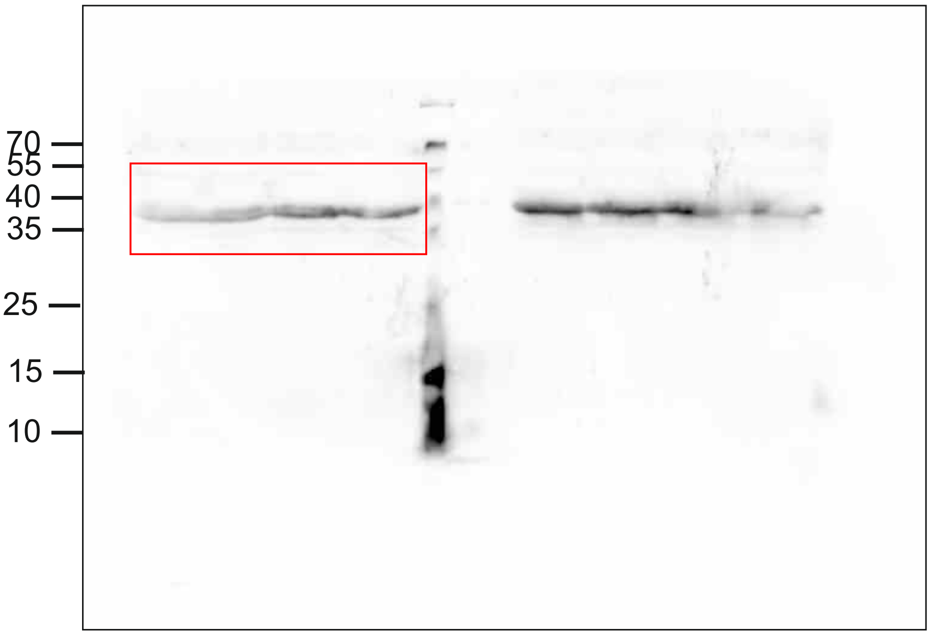

GAPDH

(B) Supplemental data for Figure 5

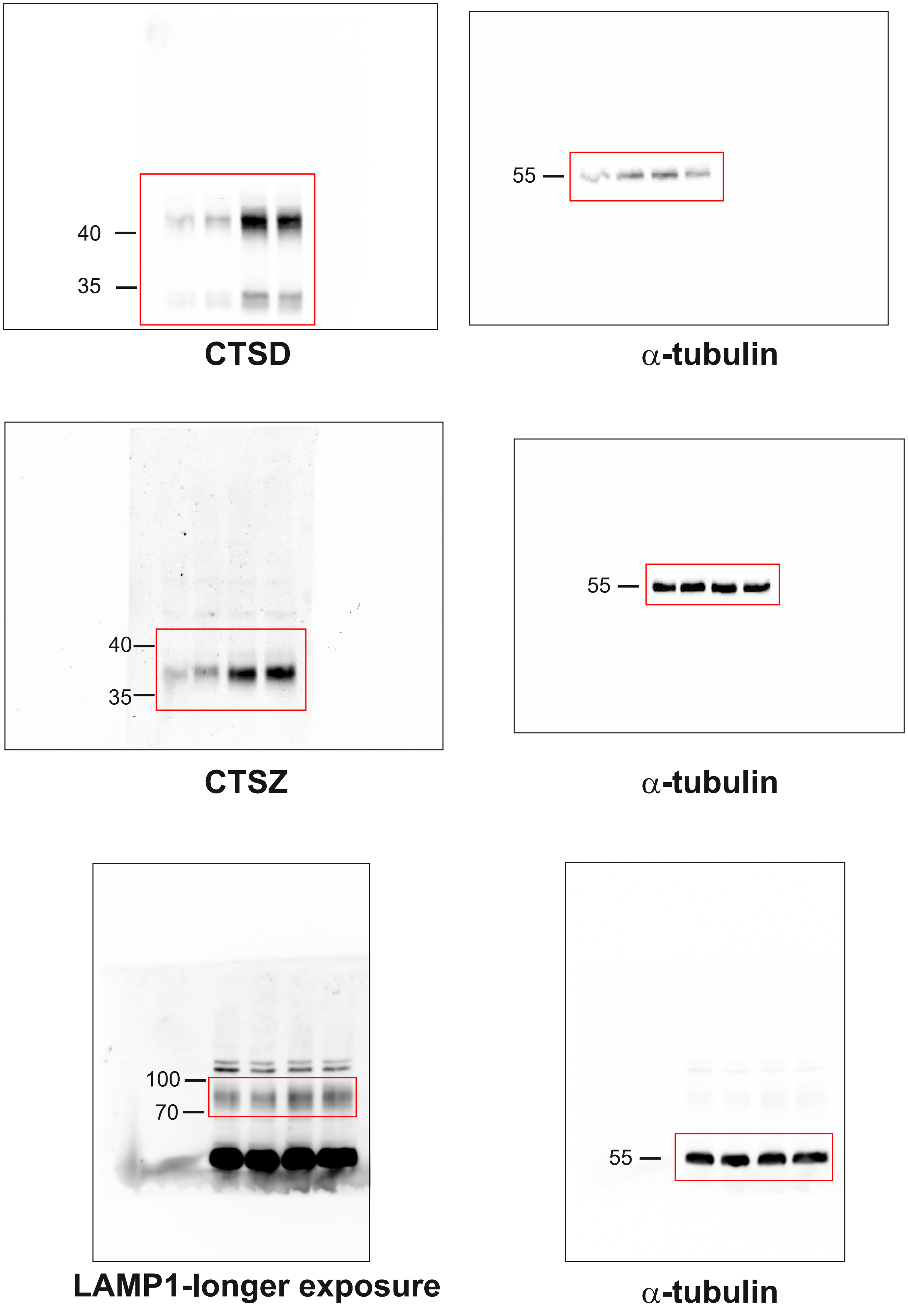

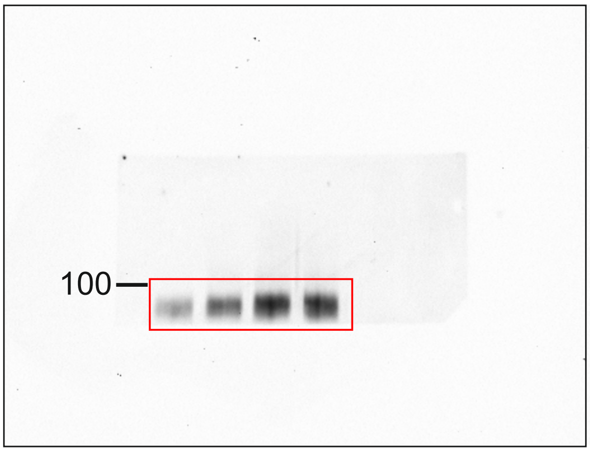

LAMP2

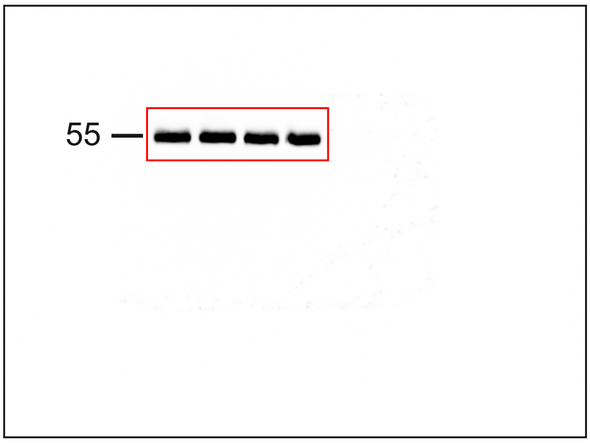

α-tubulin

(C) Supplemental data for Figure 6

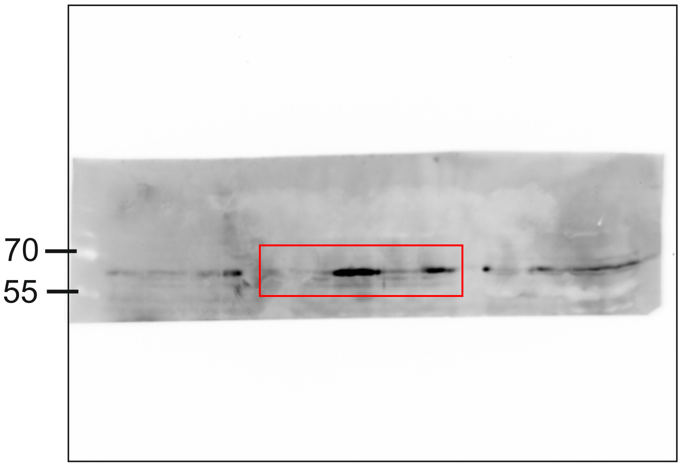

p62

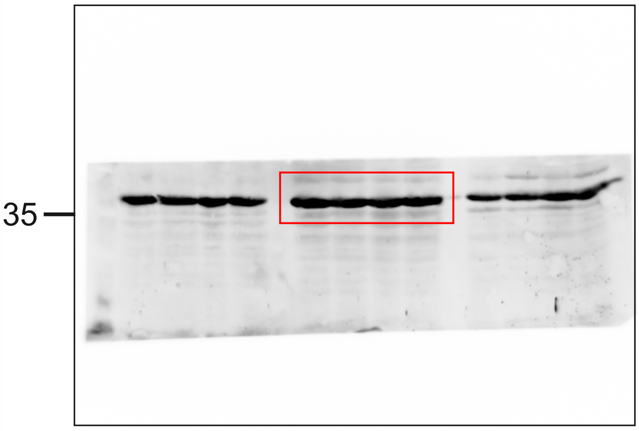

GAPDH

Supplementary Figure 5: Raw images of Western blots.

(A) Raw Western blot images used in Figure 2
(B) Raw Western blot images used in Figure 5
(C) Raw Western blot images used in Figure 6
